# Influence of Inorganic Anions on the Chemical Stability of Molybdenum Disulfide Nanosheets in the Aqueous Environment

**DOI:** 10.1021/acs.est.3c08278

**Published:** 2024-01-29

**Authors:** Ting-Wei Lee, Chiaying Chen

**Affiliations:** Department of Environmental Engineering, National Chung Hsing University, Taichung City 402, Taiwan

**Keywords:** transition metal dichalcogenides, inorganic anions, oxidative dissolution, nucleophilicity

## Abstract

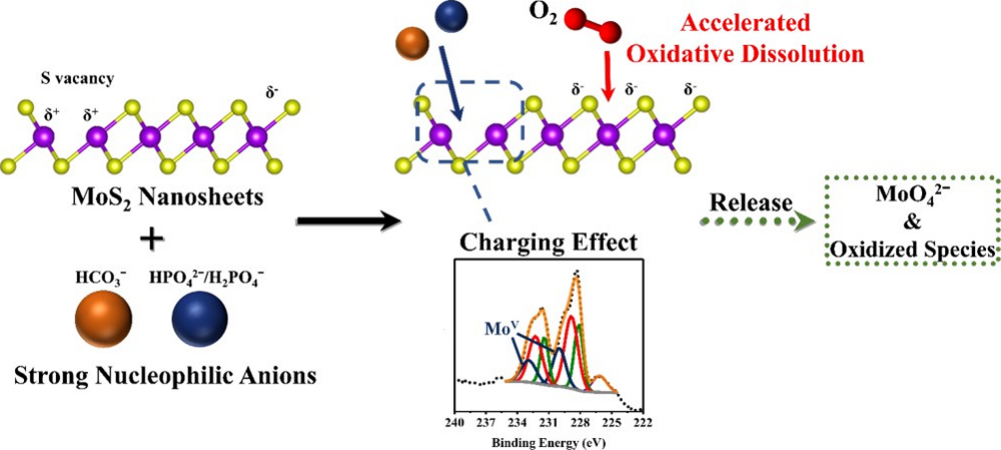

Chemical stability is closely associated with the transformations and bioavailabilities of engineered nanomaterials and is a key factor that governs broader and long-term application. With the growing utilization of molybdenum disulfide (MoS_2_) nanosheets in water treatment and purification processes, it is crucial to evaluate the stability of MoS_2_ nanosheets in aquatic environments. Nonetheless, the effects of anionic species on MoS_2_ remain largely unexplored. Herein, the stability of chemically exfoliated MoS_2_ nanosheets (ceMoS_2_) was assessed in the presence of inorganic anions. The results showed that the chemical stability of ceMoS_2_ was regulated by the nucleophilicities and the resultant charging effects of the anions in aquatic systems. The anions promote the dissolution of ceMoS_2_ by triggering a shift in the chemical potential of the ceMoS_2_ surface as a function of the anion nucleophilicity (i.e., charging effect). Fast charging with HCO_3_^–^ and HPO_4_^2–^/H_2_PO_4_^–^ was validated by a phase transition from 1T to 2H and the emergence of Mo^V^, and it promoted oxidative dissolution of the ceMoS_2_. Additionally, under sunlight, ceMoS_2_ dissolution was accelerated by NO_3_^–^. These findings provide insight into the ion-induced fate of ceMoS_2_ and the durability and risks of MoS_2_ nanosheets in environmental applications.

## Abstract 2

Oxidative dissolution of MoS_2_ in aquatic environments is affected by the nucleophilicity of anions present in the solution, which reveals the effects of inorganic anions on the fate and potential use of MoS_2_ nanosheets.

## 1 Introduction

With their remarkable attributes, two-dimensional (2D) nanosheets of layered transition metal dichalcogenides (TMDCs) have received considerable interest in both industrial and biomedical applications. Typically, the formula of a TMDC is MX_2_, where M is transition metal from groups IV, V, or VI, and X is a chalcogen such as S, Se, or Te. Among TMDCs, molybdenum disulfide (MoS_2_) is the most important 2D nanomaterial due to its tunable band gap^1^ and high electron mobility,^2^ which make MoS_2_ a promising photocatalyst, electrocatalyst, biosensor, etc. In general, MoS_2_ nanosheets exhibit three main structural phases: the tetragonal (1T) phase, hexagonal (2H) phase, and rhombohedral (3R) phase, where the 2H and 3R phases are semiconducting, and the 1T phase is metallic.^3^ Despite the utility of MoS_2_ nanosheets in practical applications, the chemical stability of the MoS_2_ nanosheets will determine their long-term applications. Prior studies probed the interaction between MoS_2_ nanosheets and environmental media, which affected the fate and transport of the MoS_2_ nanosheets. For instance, MoS_2_ nanosheets were oxidatively dissolved under alkaline^4^ and aerobic^5^ conditions. Our previous work demonstrated that the dissolution of chemically exfoliated MoS_2_ nanosheets (ceMoS_2_) was slowed in the presence of natural organic matter (NOM), including Suwannee River natural organic matter (SRNOM) and Aldrich humic acid (ALHA), in dark ambient conditions, while aging of MoS_2_ with co-occurring ALHA was accelerated by exposure to sunlight.^6^ These findings indicated the stability and behavior of MoS_2_ nanosheets in aqueous environments and were used to evaluate their persistence in the intended applications.

The interactions of MoS_2_ nanosheets with ionic species alter the characteristics and fate of the MoS_2_. For example, Li et al.^7^ showed that aggregation of MoS_2_ nanosheets dispersed by sodium cholate followed the 2D Schulze–Hardy rule, and the critical coagulation concentration (CCC) of MoS_2_ nanosheets was smaller in the presence of higher valence cations. Similarly, the CCC of MoS_2_ exposed to various cations decreased in the order K^+^ > Mg^2+^ > Al^3+^.^8^ In the presence of a natural macromolecule (e.g., NOM), the aggregation rate of MoS_2_ nanosheets was drastically reduced even with high ionic strengths (ISs).^9^ Liu et al.^10^ suggested that higher valence cations suppressed the electrical double layer for MoS_2_ nanosheets, and aggregation of the MoS_2_ nanosheets was accelerated by Ca^2+^ under visible light irradiation.^10^ In the chemisorption of cations, the sulfur atoms on MoS_2_ are soft Lewis base sites with strong affinities for soft Lewis acids (e.g., Hg^2+^ and Ag^+^).^11^ Mi and coauthors demonstrated that MoS_2_ nanosheets reduced heavy metal ions that had higher reduction potentials than MoO_4_^2–^ and SO_4_^2–^/MoS_2_ pair (0.429 V) (e.g., Ag^+^ (0.7996 V), Hg^2+^ (0.920 V), and Cr(VI) (1.232 V)) and released soluble MoO_4_^2–^ and SO_4_^2–^.^12,13^ These findings indicated that charge transfer between MoS_2_ and the cationic species altered the physical and chemical properties of MoS_2_. Nevertheless, the transformations undergone by MoS_2_ nanosheets during interaction with anionic environmental species remain largely unexplored thus far. Marks et al.^14^ illustrated the negative impact on the stability of MoS_2_ in the presence of model oxidants NO_2_^–^ and BrO_3_^–^, while dissolved oxygen is a prerequisite for the reaction. The prevalence of anions and their potential interplay with MoS_2_ have prompted exploration of the impacts of anionic species on MoS_2_ in aquatic systems.

Inorganic anionic species are ubiquitous in aqueous environments, and the concentration profiles of these anions vary with the surrounding geology, ecology, and human activities. For example, the chloride (Cl^–^) concentration in shallow groundwater has increased from 0.02 to 0.34 mM to several to tens of mM due to human activities.^15^ Fertigated water contains 1.36–4.43 mM nitrate (NO_3_^–^),^16^ and sulfate (SO_4_^2–^) concentrations of 12.09 mM originating from mining activities were found in rivers.^17^ The bicarbonate (HCO_3_^–^) present in natural water and wastewater comes from dissolved carbon dioxide in the atmosphere, with concentrations ranging from 1 to 5 mM.^18,19^ The concentration of dissolved phosphate is typically low due to its low mobility,^20^ but in swine wastewater, the total phosphorus concentration can be as high as 19.37–45.21 mM.^21^ The effluent from wastewater treatment plants (WWTPs) contains 1.30–3.36 mM Cl^–^ and 0.23–1.01 mM NO_3_^–^,^16^ and the SO_4_^2–^ concentrations in industrial effluents are 2.60–5.21 mM in most countries.^22,23^ Therefore, the engineered nanomaterials (ENMs) released or applied in water treatment facilities inevitably contact anionic species, and the efficacy of their performance could be altered by the presence of anions. Jeong et al.^24^ demonstrated that the removal of As(V) by Fe_2_O_3_ and Al_2_O_3_ was not affected by Cl^–^ or NO_3_^–^ but was inhibited by HPO_4_^2–^ due to the structural similarity of arsenate and phosphate. The removal of contaminants was minimally affected by Cl^–^, NO_3_^–^, SO_4_^2–^, and Br^–^ but was restrained by F^–^, CO_3_^2–^, PO_4_^3–^, HPO_4_^2–^, and H_2_PO_4_^–^ in graphene oxide (GO)-based composites.^25−27^ Additionally, the photocatalytic degradation of contaminants has been affected by anions. In a study by Lien et al.^28^, bromide ion (Br^–^) promoted the photocatalytic degradation of sulfamethoxazole with CaCu_3_Ti_4_O_7_ perovskite due to the formation of highly reactive radicals, while Cl^–^ decreased the degradation rate. Another study by Chen and Liu showed that higher valence anions suppressed the photodegradation efficiency, and the effect decreased in the order PO_4_^3–^ > SO_4_^2–^ > NO_3_^–^.^29^ Evidently, the effects of anions in aqueous solutions cannot be overlooked, and further research is required to fully comprehend the role of anions on ENMs. Furthermore, anions may also alter the surface characteristics and chemical stabilities of ENMs. For instance, the surfaces of silver nanoparticles (AgNPs) were chlorinated by chloride or sulfided by sulfide.^30^ Levard et al.^31^ reported that AgNPs formed solid AgCl_(s)_ at low Cl/Ag ratios (≤2675), whereas at Cl/Ag ratios ≥2675, the formation of soluble AgCl_*x*_^(*x*–1)^ led to dissolution of the AgNPs.^31^ Liu et al.^32^ demonstrated that the sulfidation of AgNPs requires O_2_. They also illustrated that direct sulfidation occurred at high sulfide levels (≥0.025 mg/L), whereas at low sulfide levels (≤0.025 mg/L), intermediate Ag^+^ species were the predominant oxidized form. A study of environmental anions by Guo et al. showed that among environmental anions, only sulfide inhibited the dissolution of AgNPs and alleviated their toxicities, but the release of Ag^+^ was not affected by phosphate or chloride.^33^ Overall, the impacts of anions on the transformations and intended applications of ENMs have been recognized; therefore, the effects of anionic species on MoS_2_ nanosheets need to be further elucidated.

Given that MoS_2_-based nanosheets are promising membrane materials for water treatment and purification, including heavy metal removal,^34^ dye rejection,^35^ and desalination,^36^ it is crucial to assess the chemical stabilities of MoS_2_ in aquatic environments with coexisting species, including anions. However, the effects of anions on MoS_2_ remain largely unknown. Thus, the aims of the present study were to elucidate the chemical stability of MoS_2_ nanosheets with coexisting environmental anions (e.g., Cl^–^, NO_3_^–^, SO_4_^2–^, HCO_3_^–^, and HPO_4_^2–^/H_2_PO_4_^–^) under dark ambient conditions and during irradiation with sunlight in aqueous environments. The effects of the anions on MoS_2_ were determined by electrochemical analyses and X-ray photoelectron spectroscopy (XPS). Our findings suggest that the presence of anionic species affected the chemical stability of the MoS_2_ nanosheets, which will enable evaluations of the roles of inorganic anions in the environmental transformations of MoS_2_.

## 2 Materials and Methods

### 2.1 Characterization and Electrochemical Measurements of ceMoS_2_

The chemicals and synthesis of ceMoS_2_ are described in the Supporting Information (Text S1 and Figure S1). The preparation of the chemically exfoliated MoS_2_ nanosheet solutions (ceMoS_2_) followed previous studies with minor adjustments.^6^ The morphology of ceMoS_2_ was surveyed by high-resolution transmission electron microscopy (TEM) (JEOL JEM-1200). The optical absorption spectrum of ceMoS_2_ was determined with a spectrophotometer (HITACHI U-3900). The concentration of the as-prepared ceMoS_2_ was determined from the absorbance at 450 nm with a mass extinction coefficient of 5010 L m^–1^ g^–1^ (Figure S2).^6^ The surfaces of ceMoS_2_ were analyzed with XPS (ULVAC-PHI, PHI 5000 VersaProbe/Scanning ESCA Microprobe). The binding energies were calibrated with the C 1s peak at 284.6 eV. The Mo 3d and S 2p core-level XPS data were analyzed with XPSPEAK41 software by using Gaussian–Lorentzian components after Shirley background subtraction. The hydrodynamic radius (*R*_h_) and zeta potential of ceMoS_2_ suspensions were measured by a ZetaSizer Nano ZS (Malvern Instrument, Worcestershire, U.K.) with a monochromatic coherent 633 nm He–Ne laser. Electron paramagnetic resonance (EPR) spectroscopy (Bruker EMX-10/12 EPR spectrometer) was applied to monitor radicals (e.g., ·OH and NO_2_·) generated from ceMoS_2_ or/and anions under a light source within the solar range (MORITEX, Hg lamp, 150 W). 5,5-Dimethyl-1-pyrroline-*N*-oxide (DMPO) was adopted as the spin-trapping agent. EPR signals were recorded at 298 K with a microwave power of 40 mW, power attenuation of 7 dB, modulation frequency of 100.0 kHz, and modulation amplitude of 1.0 G. The electrochemical investigations were conducted with an electrochemical workstation (CH Instruments, Inc.) with a three-electrode system. The glassy carbon working electrode was modified by drip-casting 10 μL of 100 mg/L ceMoS_2_ and drying at room temperature. The reference electrodes were Ag/AgCl or saturated calomel electrodes (SCE), and the counter electrode was a platinum wire. The measured potentials were converted to the reversible hydrogen electrode (RHE) potential. The probed anions (Cl^–^, NO_3_^–^, SO_4_^2–^, HCO_3_^–^, and HPO_4_^2–^/H_2_PO_4_^–^) at 100 and 10 mM were utilized as the electrolytes for the open circuit potential (OCP) and cyclic voltammetry (CV) measurements, respectively. The oxygen reduction reaction (ORR) was probed with linear scan voltammetry conducted from 0.2 to −1 V at a scan rate of 5 mV s^–1^ in O_2_-saturated electrolytes with 100 mM of the tested anions as the electrolytes.

### 2.2 Influence of Anionic Species on the Stability of ceMoS_2_

The effects of anions on ceMoS_2_ were assessed by mixing 10.5 mg/L ceMoS_2_ and varying concentrations (1, 10, and 100 mM) of different anions (Cl^–^, NO_3_^–^, SO_4_^2–^, HCO_3_^–^, and HPO_4_^2–^/H_2_PO_4_^–^) in borosilicate glass tubes and keeping them in the dark or under irradiation in an Atlas SunTest CPS+ sunlight simulator (Atlas Materials Testing Technology, Chicago, IL, USA) equipped with a 1-kW xenon arc lamp with an incident irradiance of 0.065 W/cm^2^ (Figure S3a). Throughout irradiation, the irradiated samples were maintained at 25 °C in a recirculating water bath. To probe the wavelength dependency, the effects of irradiated nitrate ions were characterized with visible wavelength illumination. ceMoS_2_ (10.5 mg/L) mixed with 10 mM NaNO_3_ was irradiated in a sunlit simulator equipped with a UV-cutoff filter that blocked irradiation with wavelengths below 420 nm (Figure S3b). For a primary assessment, the metrics of the reaction were monitored, including the absorbance at 450 nm (Abs_450_) and the pH. To further determine the effects of anions on ceMoS_2_ dissolution, the dissolved Mo species were gathered by filtering and centrifuging with ultrafiltration tubes (Amicon Ultra15 3 kDa, Millipore, USA). The concentrations of dissolved Mo species in the filtrate were digested and then determined with inductively coupled plasma–optical emission spectrometry (ICP–OES) (PerkinElmer Optima 8000). A control test indicated that there is no adsorption loss of Mo ionic species to the utilized 3 kDa MWCO membranes by comparing the concentration of sodium molybdate solution and its filtrate under different pH values and in the presence of anionic species (Figure S4). The IS effect on the dissolution of ceMoS_2_ was examined with 1, 10, and 100 mM anionic species (Cl^–^, NO_3_^–^, SO_4_^2–^, HCO_3_^–^, and HPO_4_^2–^/H_2_PO_4_^–^). It is worth noting that, except for the sets with 100 mM anions, the IS of the ionic species concentrations used in this study (Table S1) were lower than the CCC (50 mM of KCl)^37^ of ceMoS_2_. Additionally, these concentrations were also lower than the minimum level (31.6 mM of KCl)^38^ known to affect the transport of ceMoS_2_.

### 2.3 ceMoS_2_ Dissolution under Environmentally Relevant Conditions

In natural water, the concentrations of anionic species (Cl^–^, NO_3_^–^, SO_4_^2–^, HCO_3_^–^, and HPO_4_^2–^/H_2_PO_4_^–^) vary from a few μM to hundreds of mM.^15,17,39^ Although environmentally relevant concentrations of MoS_2_ are unknown, the rapid development and widespread use of MoS_2_ nanosheets has prompted the need to evaluate the transformations of MoS_2_ nanosheets. According to a prior study by Surette et al.^40^, the environmental concentrations of ENMs ranged from a few ng/L to a few mg/L, with lower concentrations considered to be more realistic. Thus, the experimental concentrations of the anions and ceMoS_2_ were 1 mM and 100 μg/L, respectively. The dissolved Mo species were collected and measured with high-resolution inductively coupled plasma–mass spectrometry (HR-ICP–MS) (Thermo Scientific Element 2), which can quantify ultralow concentrations (ng/L).

## 3 Results and Discussion

### 3.1 Characterization of ceMoS_2_

**Figure 1:**
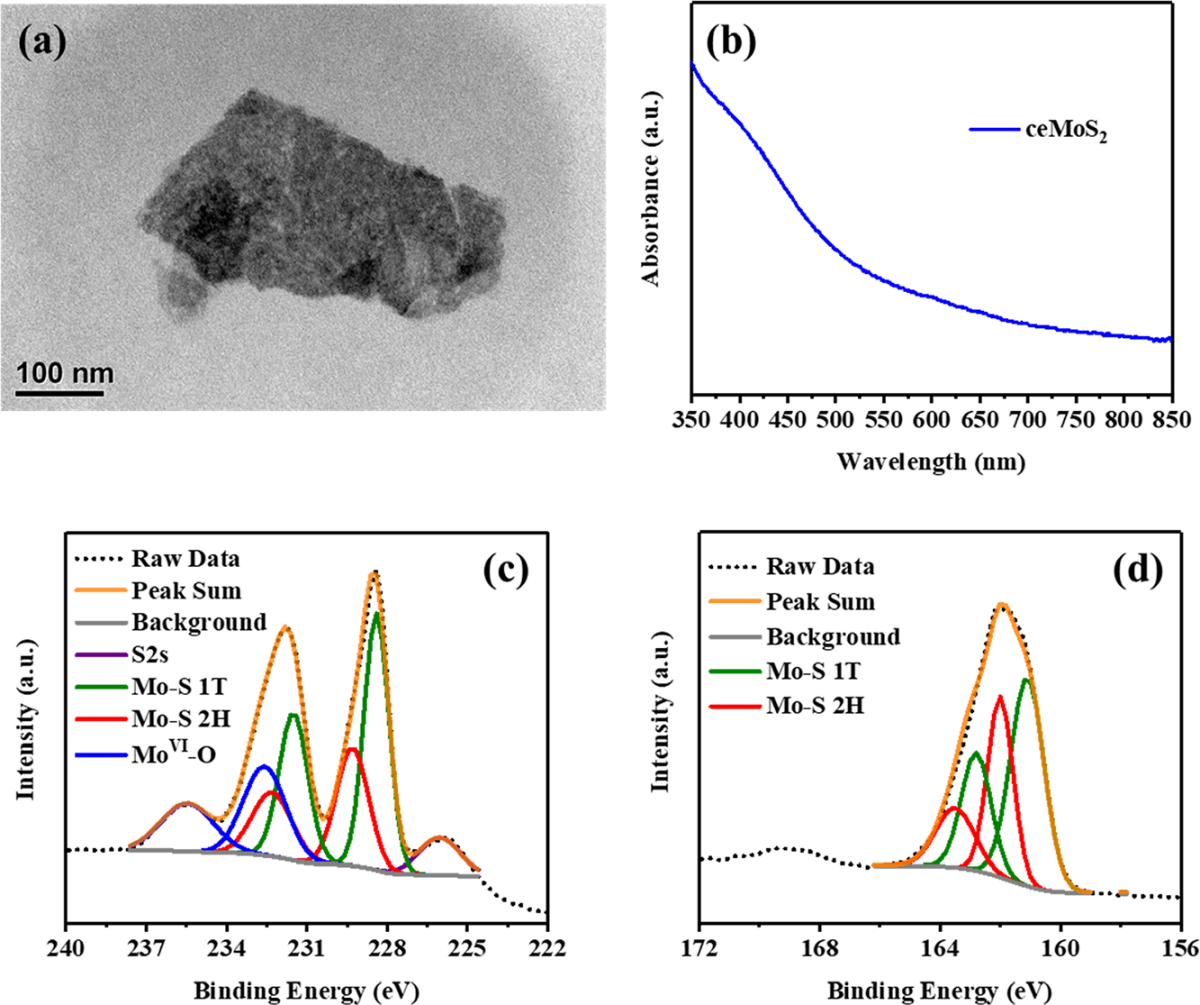
Characterization of as-prepared ceMoS_2_. (a) TEM image and (b) UV–vis absorption spectrum of ceMoS_2_. (c) Mo 3d and (d) S 2p X-ray photoelectron spectra of ceMoS_2_.

The as-prepared ceMoS_2_ was characterized with TEM, UV–vis spectrophotometry, and XPS (Figure 1). The TEM image of ceMoS_2_ (Figure 1a) showed that ceMoS_2_ had a sheet-like appearance with lateral sizes of approximately 200–300 nm, which was consistent with the general morphology of ceMoS_2_.^4^ In the UV–vis spectrum (Figure 1b), ceMoS_2_ exhibited no peaks in the visible region, which was attributed to the metallic nature of the 1T phase,^41^ the dominant phase in the chemically exfoliated (i.e., lithium-intercalated) MoS_2_ nanosheets. The orbital configuration and phase composition of ceMoS_2_ were analyzed by XPS. In Figure 1c, the Mo 3d doublets of ceMoS_2_ were located at approximately 229.0 eV (Mo 3d_5/2_). After peak fitting, the Mo 3d peaks of ceMoS_2_ were deconvoluted into the 1T phase (3d_5/2_: 228.2 eV, 3d_3/2_: 231.3 eV) and 2H phase (3d_5/2_: 229.1 eV, 3d_3/2_: 232.2 eV), which indicated the different binding energies (0.7–0.9 eV) of the 1T and 2H phases.^42^ The 1T phase content in the Mo–S bond was 64.7%, confirming the predominance of the 1T phase in ceMoS_2_. In the S 2p core-level spectrum (Figure 1d), the broad peak was partitioned into four peaks corresponding to the 1T phase (2p_3/2_: 161.1 eV, 2p_1/2_: 162.8 eV) and the 2H phase (2p_3/2_: 162.0 eV, 2p_1/2_: 163.5 eV). The content of the 1T phase indicated by the S 2p XPS data was 64.1%, which was similar to the result from the Mo 3d XPS data. The laminate structure and the presence of the 1T phase in the as-prepared ceMoS_2_ implied a successful synthesis of chemically exfoliated MoS_2_.

### 3.2 Effect of Anions on the Suspension Stability and Dissolution of ceMoS_2_

The effects of anions on the chemical stability of ceMoS_2_ were probed in the presence of 1–20 mM NaCl, considering that Cl^–^ is one of the most common anions in both natural water^43^ and wastewater.^44^ Given the correlation between the absorbance at 450 nm and the concentration of suspended MoS_2_ (Figure S2), the changes in the concentration of MoS_2_ nanosheets were tracked with the normalized absorbance *A*_*t*_/*A*_0_, where *A*_*t*_ and *A*_0_ are the 450 nm absorbance of the ceMoS_2_ dispersion at time *t* and the initial point, respectively. As shown in Figure S5, a slight decrease in *A*_*t*_/*A*_0_ was observed upon increasing the Cl^–^ concentration from 1 to 20 mM under dark conditions. During exposure to sunlight, the decrease in *A*_*t*_/*A*_0_ for ceMoS_2_ became pronounced at higher chloride concentrations (10 and 20 mM), which were 2.0 and 2.8 times greater than that of the control at 48 h, indicating light-accelerated destabilization of the ceMoS_2_ dispersion. The light-induced destabilization of MoS_2_ near the surface of the sunlit water was ascribed to the surface plasmon oscillations enhanced by the cations (e.g., Na^+^),^10^ which decreased the energy barrier for aggregation.

Additionally, the concentration of dissolved Mo species produced after 72 h was determined by passing the suspensions through 3 kDa membranes. The dissolution rate of ceMoS_2_ increased from 29.1% (0 mM Cl^–^) to 35.2% (1 mM Cl^–^), 40.1% (5 mM Cl^–^), 42.9% (10 mM Cl^–^), and 54.3% (20 mM Cl^–^) in the dark, and the dissolution rates were increased to 47.4% (0 mM Cl^–^) and 68.0% (20 mM Cl^–^) with light exposure. The concurrent dissolution of Mo species and the decrease in *A_t_*/*A*_0_ suggested that the destabilization of ceMoS_2_ with Cl^–^ and light was due to not only light- and cation-induced aggregation but also oxidative dissolution of the ceMoS_2_. Furthermore, the effects of different anions on the transformations of ceMoS_2_ and the role of sunlight have not been well-explored previously. Therefore, the transformations of ceMoS_2_ dispersions were examined with various anions (i.e., Cl^–^, SO_4_^2–^, NO_3_^–^, HCO_3_^–^, and HPO_4_^2–^/H_2_PO_4_^–^) found in aquatic systems.

**Figure 2:**
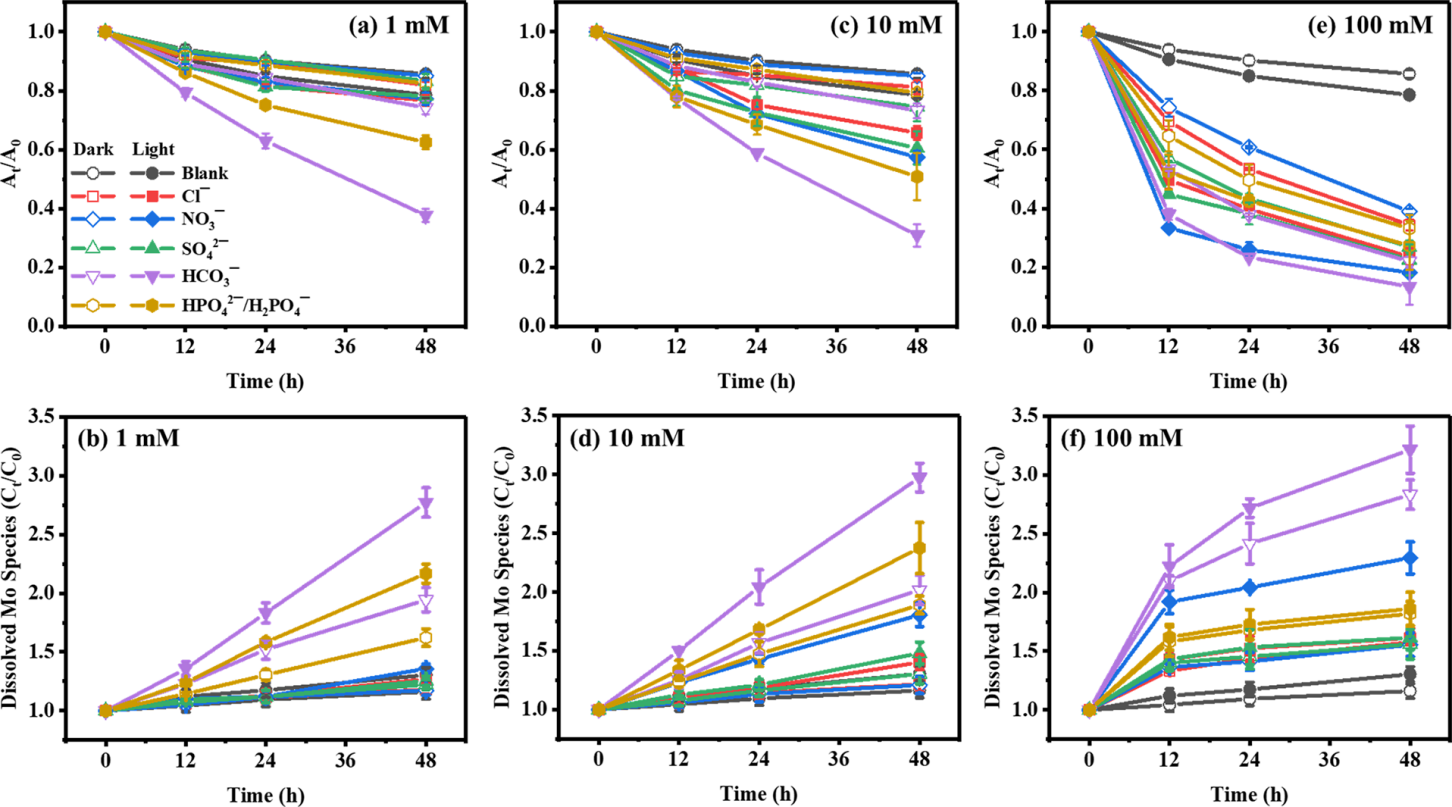
Stabilities of ceMoS_2_ suspensions (10.5 mg/L) determined by measuring the normalized absorbance at 450 nm (*A*_*t*_/*A*_0_) and the normalized concentration of dissolved Mo species (passed through 3 kDa membranes) (*C*_*t*_/*C*_0_) in the presence of (a,b) 1 mM, (c,d) 10 mM, and (e,f) 100 mM anionic species under both dark and solar irradiation. Error bars are sample standard deviations from triplicate measurements.

The stabilities of the ceMoS_2_ dispersions were probed in the presence of various anions with concentrations of 1, 10, and 100 mM. As shown in Figure 2a, a faster decrease in *A_t_*/*A*_0_ over time was found in the presence of 1 mM HCO_3_^–^ and HPO_4_^2–^/H_2_PO_4_^–^ under both dark and irradiation conditions. Notably, with the elimination of O_2_, the stabilities of ceMoS_2_ suspensions in the presence of the probed anions exhibited no significant difference to the blank control (Figure S6), illustrating the essential role of O_2_ in the promoted decrease in *A_t_*/*A*_0_ of MoS_2_ by HCO_3_^–^ and HPO_4_^2–^/H_2_PO_4_^–^. During irradiation with sunlight (solid symbols), the *A*_*t*_/*A*_0_ ratios for ceMoS_2_ declined to 0.62 and 0.38 in the presence of 1 mM HPO_4_^2–^/H_2_PO_4_^–^ and HCO_3_^–^, respectively. To determine the oxidative transformation of ceMoS_2_ caused by anions, the products from ceMoS_2_ were identified by filtering the dispersions through 3 kDa MWCO membranes, and the dissolved Mo species from ceMoS_2_ were monitored after incubation with anions. As shown in Figure 2b, the amount of ionic Mo species produced (*C*_*t*_/*C*_0_) was computed from the normalized concentration of ionic Mo species at a predetermined time (*C*_*t*_) and the initial concentration of dissolved Mo species (*C*_0_) (i.e., 2.65 mg/L). The produced ionic Mo species displayed a similar trend for *A*_*t*_/*A*_0_ and illustrated that HCO_3_^–^ and HPO_4_^2–^/H_2_PO_4_^–^ facilitated the formation of dissolved ionic species from ceMoS_2_ under both dark and irradiated conditions. Note that the MoS_2_ nanosheets can produce photogenerated free radicals (e.g., ·O_2_^–^ and ·OH), leading to facilitated oxidative dissolution of MoS_2_ with light exposure.^5^ Furthermore, bicarbonate and phosphate promoted the detachment of photogenerated reactive radicals from the surfaces of materials, thereby increasing the mobility of radicals and enhancing the oxidation reaction.^45,46^ As shown in Figure S7, photogenerated ·OH was identified in the irradiated ceMoS_2_ suspension in the presence of HCO_3_^–^ and HPO_4_^2–^/H_2_PO_4_^–^, suggesting that the further oxidative degradation of ceMoS_2_ promoted by HCO_3_^–^ and HPO_4_^2–^/H_2_PO_4_^–^ under irradiation was attributed to the enhanced availability of the photogenerated active species.

In the presence of a higher concentration of anions (10 mM in Figure 2c), *A*_*t*_/*A*_0_ showed a greater decline rate compared to those seen at 1 mM, particularly under irradiation, which was due to the destabilization of ceMoS_2_ during irradiation in concentrated electrolyte solutions. The light-induced destabilization of ceMoS_2_ was further verified by the zeta potential of ceMoS_2_ suspensions (Figure S8), which illustrates that the zeta potential of irradiated ceMoS_2_ with anionic species became less negative (i.e., destabilization) as a function of time, while the zeta potential remained relatively unchanged under dark condition. In Table 1, the amount of ionic Mo species produced (*C*_*t*_/*C*_0_) was further computed from the normalized concentration of ionic Mo species at longer term for 72 h (*C_t_*) and the initial concentration of dissolved Mo species (*C*_0_). In the presence of HCO_3_^–^ and HPO_4_^2–^/H_2_PO_4_^–^, the production of ionic Mo species from ceMoS_2_ was significantly elevated under both dark and irradiated conditions. Additionally, greater dissolution of ceMoS_2_ was found with NO_3_^–^ and irradiation. The findings were consistent with observed *A*_*t*_/*A*_0_, demonstrating that the chemical stability of the MoS_2_ nanosheets was affected by the different anions in aquatic systems.

**Table 1:** Concentrations of Ionic Mo Species (Obtained by Passage through 3 kDa Membranes) in ceMoS_2_ (10.5 mg/L) Incubated with 10 mM Anions for 72 h

coexisting anions	ionic Mo species at 0 h	ionic Mo species at 72 h in dark		ionic Mo species at 72 h under sunlight irradiation	
	*C*_0_ (mg/L)	*C*_*t*_ (mg/L)	*C*_*t*_/*C*_0_	*C*_*t*_ (mg/L)	*C*_*t*_/*C*_0_
blank	2.65 ± 0.11	3.35 ± 0.13	1.26 ± 0.08	3.86 ± 0.14	1.46 ± 0.09
blank (visible light)				3.77 ± 0.13	1.42 ± 0.08
Cl^–^		3.82 ± 0.20	1.44 ± 0.11	4.31 ± 0.22	1.63 ± 0.12
SO_4_^2–^		3.85 ± 0.17	1.45 ± 0.10	4.65 ± 0.13	1.75 ± 0.10
NO_3_^–^		3.60 ± 0.16	1.36 ± 0.09	6.29 ± 0.19	2.37 ± 0.14
NO_3_^–^ (visible light)				4.10 ± 0.12	1.55 ± 0.09
HCO_3_^–^		6.19 ± 0.27	2.33 ± 0.16	10.49 ± 0.34	3.96 ± 0.24
HPO_4_^2–^/H_2_PO_4_^–^		5.82 ± 0.18	2.19 ± 0.13	7.81 ± 0.21	2.95 ± 0.16

With 100 mM anion concentrations (Figure 2e), the *A_t_*/*A*_0_ for ceMoS_2_ plummeted during the first 12 h, indicating that the suspension stability was disrupted at high anion concentrations. The suspension stability was monitored using time-resolved dynamic light scattering (DLS) to determine the time-dependent change in the hydrodynamic radius (*R*_h_). Although DLS is not ideal for determining nonspherical particles, the intensity averaged *R*_h_ could be used to obtain a general index of the size population of ceMoS_2_ suspensions.^10,47^ As shown in Figure S9, the significant increase in *R*_h_ with the presence of 100 mM anionic species confirms the disrupted suspension stability at higher anion concentrations. In the dissolved Mo species measurement, an elevated release of Mo ionic species was seen with 100 mM HCO_3_^–^ and HPO_4_^2–^/H_2_PO_4_^–^ and was increased by 129.3% with NO_3_^–^ and irradiation for 48 h (Figure 2f). The nitrate-enhanced transformations of ceMoS_2_ seen during solar irradiation are discussed later. Collectively, the findings revealed that oxidative transformation of the ceMoS_2_ was promoted to different degrees by environmental anions; HCO_3_^–^ and HPO_4_^2–^/H_2_PO_4_^–^ promoted the degradation of ceMoS_2_ into dissolved Mo species under both dark and irradiated conditions, while NO_3_^–^ exhibited light-accelerated dissolution of ceMoS_2_ at higher concentrations.

It is worth noting that the trend for oxidative dissolution was clear in the dissolved Mo species measurements, while *A*_*t*_/*A*_0_ determined the concentration of the MoS_2_ dispersion, which was affected by aggregation and sedimentation of the ceMoS_2_ nanosheets. As listed in Table S2, the time-dependent *A*_*t*_/*A*_0_ in 1, 10, and 100 mM anionic species in Figure 2 was fit by the first-order decay equation, which illustrates an increase in rate constants under higher anion concentrations (e.g., 100 mM) and light exposure. Given that the aggregation and sedimentation of the ceMoS_2_ nanosheets contribute to the normalized absorbance *A*_*t*_/*A*_0_,^10^ the results, in addition to the zeta potential (Figure S8) and DLS measurement (Figure S9), clearly indicated a light- and higher-anion-concentration-induced destabilization of ceMoS_2_. The suspension stability is known to be one of the factors affecting the dissolution of ENM particles.^48−50^ As shown in Figure 2b,d, the dissolved Mo species produced in ceMoS_2_ were comparable with 1 and 10 mM anionic species, while ceMoS_2_ with 100 mM anionic species demonstrated a higher initial dissolution rate (Figure 2f). Along with the potential influence of aggregation on ceMoS_2_ dissolution at higher anion concentrations, the promoted oxidative dissolution of ceMoS_2_ by HCO_3_^–^ and HPO_4_^2–^/H_2_PO_4_^–^, as well as irradiated NO_3_^–^, was consistent in the probed anion concentration range (i.e., 1–100 mM).

Most of the concentrations of ionic Mo species were higher in the sunlight-irradiated samples than in the dark samples (Table 1), while irradiation with NO_3_^–^ produced more dissolved Mo species (*C_t_*/*C*_0_ = 2.37). The enhancement of ceMoS_2_ dissolution by NO_3_^–^ and sunlight was attributed to photolysis of the nitrate ions and the generation of hydroxyl radicals:^51,52^
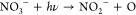
1
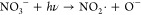
2
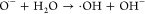
3

**Scheme 1:**
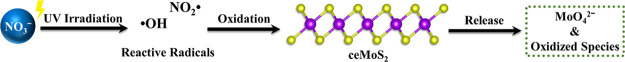
UV-Accelerated Oxidative Dissolution of ceMoS_2_ with Nitrate Anions

EPR analysis was employed to determine the photoproduced radicals. In Figure S10a, the photogenerated ·OH and NO_2_· in NO_3_^–^ were detected by distinguishable EPR signals of 2-hydroxy-5,5-dimethyl-1-pyrrolidinyloxy (DMPO–OH)^53^ and 5,5-dimethyl-2-oxopyrroline-1-oxyl (DMPOX)^54^ (Figure S10b) adducts, respectively. Notably, the oxidation of ceMoS_2_ by nitrate was more pronounced under sunlight irradiation. In a comparison experiment, the *C*_*t*_/*C*_0_ of Mo ionic species with NO_3_^–^ and visible light irradiation (with UV-cutoff filter (<420 nm)) was 1.55, which was much lower than that seen for full-spectrum sunlight irradiation (i.e., *C*_*t*_/*C*_0_ = 2.37). This was consistent with the fact that NO_3_^–^ absorbs light below 350 nm (Figure S11) in the solar irradiation,^51^ while other anions absorbed no UV and were optically transparent. Scheme 1 depicts the UV-accelerated oxidation and dissolution of ceMoS_2_ with nitrate anions.

Given that the greatest oxidative dissolution of ceMoS_2_ was found in the presence of HCO_3_^–^, the ionic Mo species produced in the oxidative dissolution process were examined by ion chromatography. As illustrated in Figure S12, the MoO_4_^2–^ and SO_4_^2–^ produced indicated that aging of the ceMoS_2_ in the presence of HCO_3_^–^ occurred according to the reported dissolution reaction of MoS_2_ (eq 4),^4^ which suggested that oxidative dissolution, rather than the complexation of anion by the ionic Mo species, was likely the dominant reaction pathway.
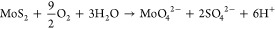
4

Previously, NO_2_^–^ and BrO_3_^–^ acted as the oxidants for promoting the dissolution of MoS_2_ in the presence of dissolved oxygen, owing to their relative ease of reduction. Given that the reduction potential of the probed anions in the current work is generally more negative (see Table S3) than that of MoO_4_^2–^ and SO_4_^2–^/MoS_2_ pair (0.429 V),^12^ the probed anions herein were likely not acting as oxidants. While the essential role of O_2_ in this oxidative dissolution has been illustrated (Figure S6), the promoted oxidative dissolution of ceMoS_2_ by HCO_3_^–^ and HPO_4_^2–^/H_2_PO_4_^–^ is surprising since they are unlikely to act as oxidants, thereby further investigation through electrochemical characterization will be conducted later to unveil the origin of the enhanced oxidation. Note that the ionic radius of SO_4_^2–^ and H_2_PO_4_^–^ are larger than Cl^–^, NO_3_^–^, HPO_4_^–^, and HCO_3_^–^ (Table S4), which results in no identifiable correlation with the observed trend in the oxidative dissolution of ceMoS_2_ and thus suggests that the ionic radius did not likely play a decisive role in affecting the oxidative dissolution of MoS_2_. Additionally, along with the dissolved Mo species, the protons released throughout the aging process were monitored. As demonstrated in Figure S13, the pH of the ceMoS_2_ solution decreased from neutral to acidic in the presence of Cl^–^, NO_3_^–^, and SO_4_^2–^, but the pH was relatively stable in the presence of HCO_3_^–^ and HPO_4_^2–^/H_2_PO_4_^–^, which was likely due to their buffering capacities. Notably, a pH dependence was previously illustrated for oxidative dissolution of ceMoS_2_, and the dissolution rates were accelerated at higher pH.^4^ To examine whether the oxidative dissolution of ceMoS_2_, particularly in the presence of HCO_3_^–^, resulted solely from pH-induced reactions, the *A*_*t*_/*A*_0_ values for ceMoS_2_ with HCO_3_^–^ and ceMoS_2_ in the control medium (i.e., no anions) were compared at pH 8.5. In Figure S14, the pronounced *A*_*t*_/*A*_0_ declines seen under both dark and light conditions in the presence of HCO_3_^–^ compared to those in the pH 8.5 control medium, clearly indicated that other factors, in addition to the pH dependency, regulated the oxidative dissolution of MoS_2_. The mechanism for dissolution of ceMoS_2_ in the presence of anions is discussed later.

### 3.3 Morphology and Phase Transitions of ceMoS_2_ Triggered by Anions

**Figure 3:**
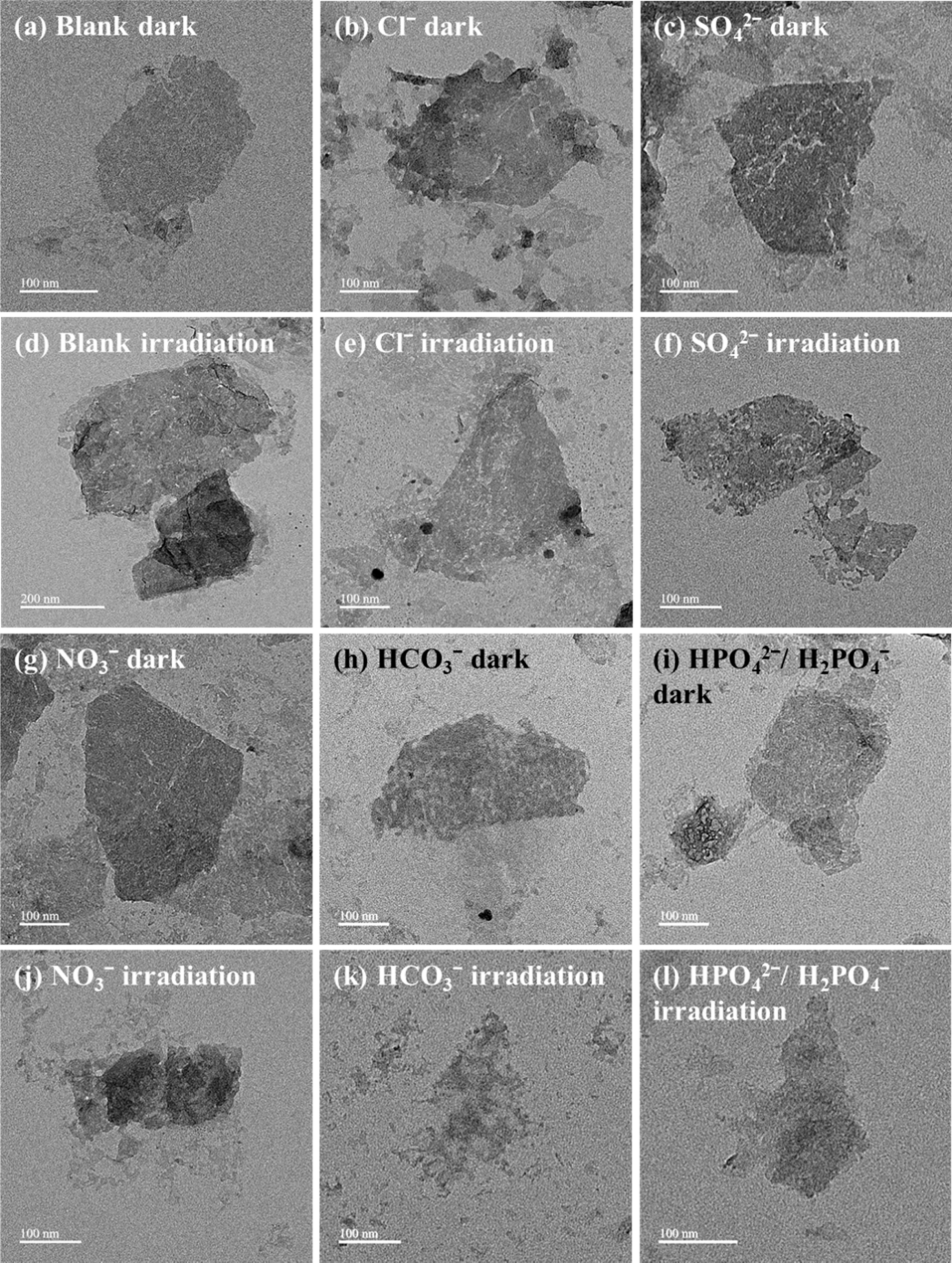
TEM images of ceMoS_2_ (10.5 mg/L) incubated for 72 h in 10 mM anion solutions. Under dark conditions: (a) blank, (b) Cl^–^, (c) SO_4_^2–^, (g) NO_3_^–^, (h) HCO_3_^–^, and (i) HPO_4_^2–^/H_2_PO_4_^–^. Under irradiation: (d) blank, (e) Cl^–^, (f) SO_4_^2–^, (j) NO_3_^–^, (k) HCO_3_^–^, and (l) HPO_4_^2–^/H_2_PO_4_^–^.

The morphological and phase transitions of ceMoS_2_ triggered by anions were analyzed by TEM and XPS. As shown in Figure 3a, no change in the as-prepared nanosheets (Figure 1a) was observed after 72 h of dark incubation. In the presence of anions under dark conditions, the structure of the MoS_2_ nanosheets deteriorated, and cracks appeared on the surface and edges, particularly in the presence of HCO_3_^–^ (Figure 3h) and HPO_4_^2–^/H_2_PO_4_^–^ (Figure 3i). The anion-induced morphological deterioration was consistent with the ceMoS_2_ dissolution profiles, both of which indicated the decay of ceMoS_2_ stability. During irradiation, no significant difference was observed with Cl^–^ (Figure 3e) and SO_4_^2–^ (Figure 3f) relative to their dark controls. In contrast, the sheet edges of ceMoS_2_ were destroyed by the presence of NO_3_^–^ (Figure 3j), indicating photoenhanced destruction by nitrate. In the presence of HCO_3_^–^ (Figure 3k), ceMoS_2_ was utterly fragmented, which was consistent with the enhanced dissolution of ceMoS_2_ during sunlight exposure (Figure 2).

**Figure 4:**
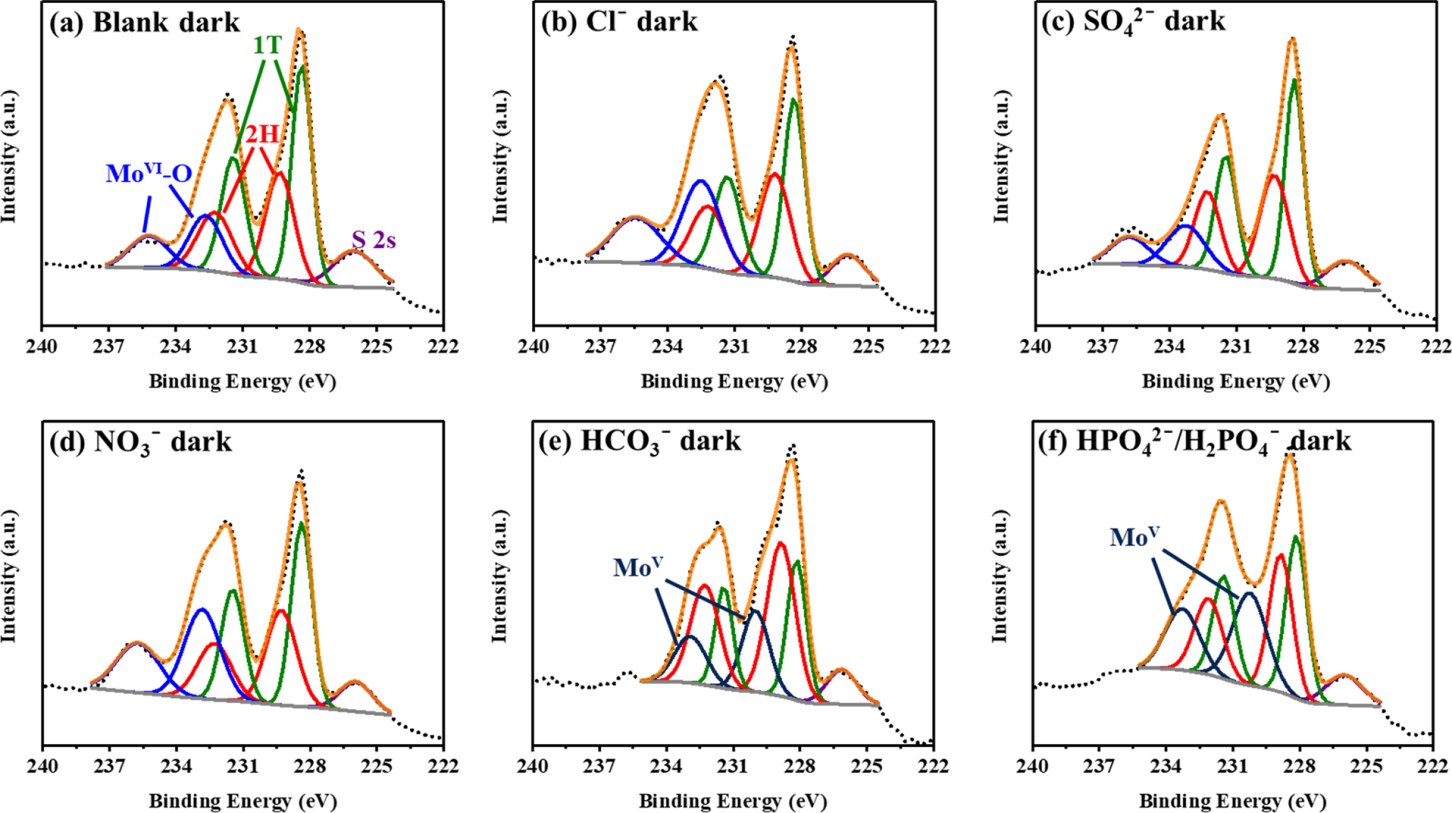
Mo 3d core-level XPS data for ceMoS_2_ (10.5 mg/L) incubated with 10 mM anions under dark conditions: (a) blank, (b) Cl^–^, (c) SO_4_^2–^, (d) NO_3_^–^, (e) HCO_3_^–^, and (f) HPO_4_^2–^/H_2_PO_4_^–^. The Mo 3d core-level spectra of the sunlight-illuminated samples and the fitting data in the dark or under sunlight are shown in Figure S15.

Prior studies showed phase-dependent oxidative dissolution of ceMoS_2_ during the aging process; the 1T phase selectively underwent oxidative dissolution, leading to conversion of the 1T phase to the 2H phase.^4,6^ Herein, the phase transition of ceMoS_2_ was monitored in the presence of various anions with high-resolution Mo 3d core-level XPS under both dark and light conditions (Figures 4 and S15). After a 72-h incubation in the dark (Figure 4a), the 1T content of ceMoS_2_ exhibited a slight decline from 64.7% (as-prepared, Figure 1c) to 58.7% in the dark blank and 55.4% under sunlight. In addition to the phase transition, an increase in the Mo^VI^–O content of ceMoS_2_ was observed from 19.8% in the dark to 30.9% under irradiation. In the presence of Cl^–^, NO_3_^–^, and SO_4_^2–^, ceMoS_2_ displayed 1T:2H ratios similar to that in the blank, which was consistent with the relatively low dissolution rate of ceMoS_2_ in the presence of these three anions. On the other hand, for HCO_3_^–^ and HPO_4_^2–^/H_2_PO_4_^–^, the 1T proportions declined to 38.0 and 51.4%, respectively, indicating that HCO_3_^–^ and HPO_4_^2–^/H_2_PO_4_^–^ promoted the phase transition (1T to 2H) of ceMoS_2_. Additionally, the hexavalent Mo (Mo^VI^–O) in ceMoS_2_ disappeared with HCO_3_^–^ and HPO_4_^2–^/H_2_PO_4_^–^, and new peaks were found at approximately 230 eV (3d_5/2_) and 233 eV (3d_3/2_), for pentavalent Mo (Mo^V^).^55^ The origin of the emergence of Mo^V^ with HCO_3_^–^ and HPO_4_^2–^/H_2_PO_4_^–^ will be discussed later. Under sunlight irradiation for 72 h, the proportion of the 1T phase in ceMoS_2_ exposed to all anions was decreased to a lower level compared to that in the dark. In particular, the Mo^VI^–O proportion reached its highest value (62.0%) in the presence of NO_3_^–^ and sunlight irradiation, suggesting that a photoreaction of NO_3_^–^ with ceMoS_2_ caused a greater oxidation of the 1T phase. The photooxidation of ceMoS_2_ by NO_3_^–^ was likely due to the formation of reactive radicals that oxidized the MoS_2_ nanosheets (Scheme 1). The changes in morphology and phase transition indicated that the stability of ceMoS_2_ varied as a function of anion species and light exposure, and detailed mechanisms are discussed in the following section.

### 3.4 Dissolution Mechanism for ceMoS_2_ in the Presence of Anions

**Figure 5:**
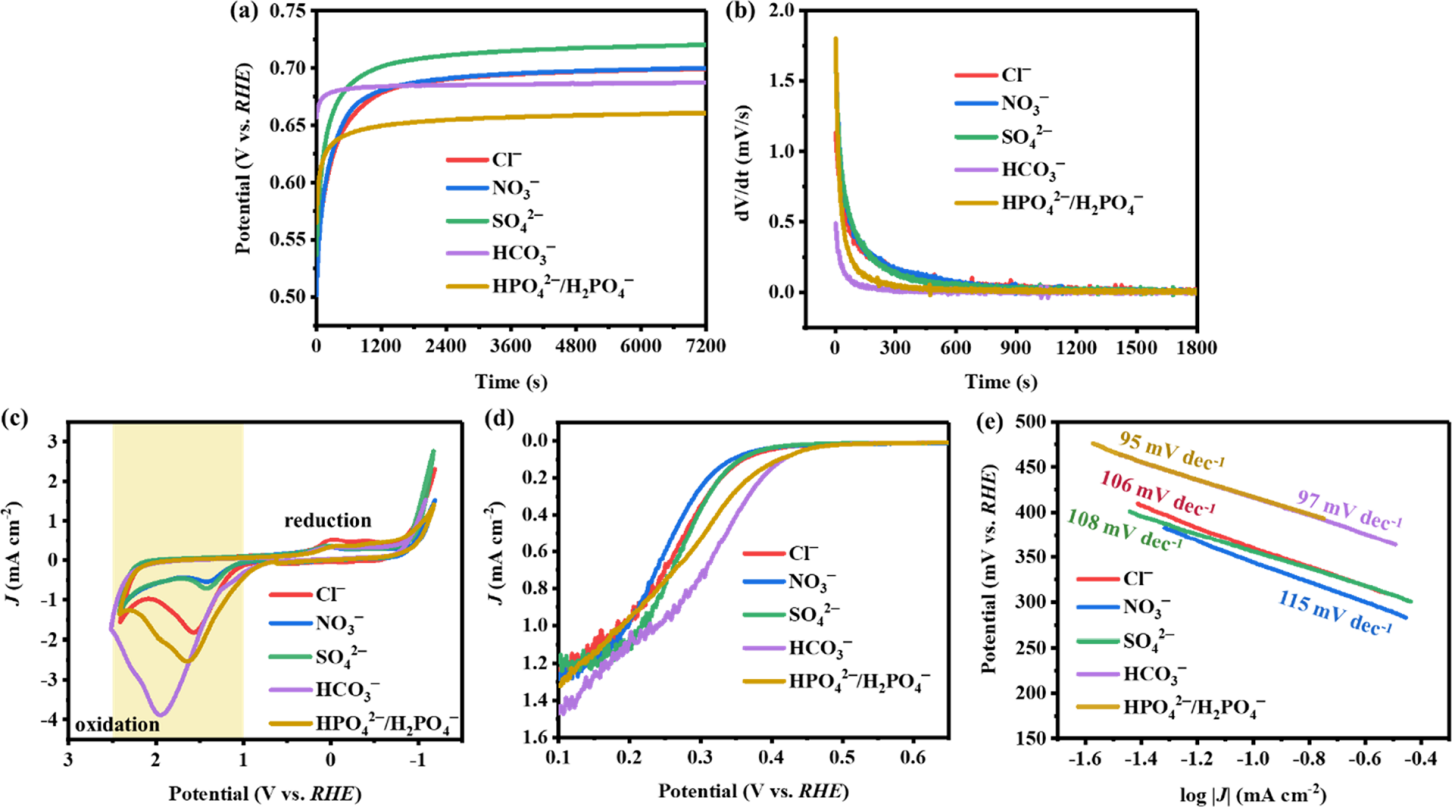
Electrochemical analyses of ceMoS_2_ in the presence of anionic electrolytes (Cl^–^, NO_3_^–^, SO_4_^2–^, HCO_3_^–^ and HPO_4_^2–^/H_2_PO_4_^–^); (a) OCP evolution of ceMoS_2_ in the presence of anions and (b) the OCP kinetics of ceMoS_2_ in the presence of anions (electrolyte: 100 mM of anions). (c) CV curves for ceMoS_2_ in the presence of anions (electrolyte: 10 mM of anions). (d) ORR polarization curves and (e) corresponding Tafel slopes for ceMoS_2_ in the presence of different anions (electrolyte: 100 mM of anions).

To elucidate the interactions between ceMoS_2_ and anions, the electrochemistry of ceMoS_2_ was probed with various approaches, including OCP, CV, and ORR. As described by Lee et al.^56^, during the OCP process, the electrons transferred from the electrolyte charged the electrode surface. Thus, with a shorter time required to achieve a stable potential, electron transfer from the anions to the electrode surface is accelerated. In Figure 5a, the OCP of ceMoS_2_ in a 100-mM anion solution was recorded for 2 h to reach a stable potential. As shown in Figure 5b, the OCP stabilization rate of ceMoS_2_ was faster with HCO_3_^–^ and HPO_4_^2–^/H_2_PO_4_^–^, while the rates with Cl^–^, SO_4_^2–^, and NO_3_^–^ were relatively slow. Given that there was no current flowing during OCP measurements (i.e., equilibrium was achieved between the working electrode and the reference electrode in the electrolyte),^57^ the OCP change indicated the electron transfer rate from the electrolyte to the electrode surface. Therefore, HCO_3_^–^ and HPO_4_^2–^/H_2_PO_4_^–^ exhibited faster charging rates with ceMoS_2_, while Cl^–^, SO_4_^2–^, and NO_3_^–^ exhibited slower charging rates. Fast electron charging with the HCO_3_^–^ and HPO_4_^2–^/H_2_PO_4_^–^ was confirmed by the emergence of Mo^V^ (Figure 4e,f), rather than the transition to Mo^VI^, in the presence of other anions.

Next, the extent of ceMoS_2_ oxidation by the different anions was assessed by CV. Figure 5c shows the oxidation sweep starting at 0 V followed by the reduction sweep. The oxidation peaks indicated oxidation of the ceMoS_2_ nanosheets, while the reduction peaks were attributed to reduction of the oxidized MoS_2_.^58,59^ Among the anionic electrolytes, HCO_3_^–^ and HPO_4_^2–^/H_2_PO_4_^–^ generated higher oxidation peak currents for ceMoS_2_, implying oxidation of more ceMoS_2_. Additionally, the reduction peak currents for ceMoS_2_ were negligible in the presence of HCO_3_^–^ and HPO_4_^2–^/H_2_PO_4_^–^, which was ascribed to oxidative dissolution of the ceMoS_2_. This led to deterioration of the electrode material (i.e., ceMoS_2_) and explained the low reduction peak current in the subsequent cathodic scan.^60^ Lower current intensities for ceMoS_2_ were observed in the presence of NO_3_^–^, SO_4_^2–^, and Cl^–^, suggesting less ceMoS_2_ was oxidized. The electrochemical data were consistent with the observed trend for the dissolution of ceMoS_2_ in anion solutions under dark conditions (Table 1).

The results of the OCP and CV suggested different charging rates and oxidative dissolution of ceMoS_2_ in the presence of the examined anions, which was ascribed to the chemical potential of the ceMoS_2_ surface. In a prior study, Lenhart and coauthors^61^ indicated that the oxidative dissolution of AgNPs by electron acceptors (e.g., O_2_) was catalyzed by nucleophilic reagents owing to alteration of the chemical potential of the AgNPs by the nucleophiles. In the absence of nucleophiles, oxidation of the AgNPs shifted the chemical potential of the particle surfaces to more positive values, which decreased the difference in the chemical potential between the AgNPs and the electron acceptors (e.g., O_2_) and slowed the oxidation. In contrast, in the presence of absorbed nucleophiles, the nucleophilic reagents generated excess negative charge^62^ and shifted the chemical potential of the AgNPs to a more negative value, enabling oxidation of the AgNPs. A nucleophile is an electron-rich molecule or ion with a lone pair of electrons, and it reacts with electron-deficient compounds;^63^ the anions used in this study are nucleophiles with different nucleophilicities. The nucleophilic constants of HCO_3_^–^, HPO_4_^2–^, Cl^–^, SO_4_^2–^, and NO_3_^–^ are 3.8, 3.8, 2.7, 2.5, and 1.0,^64,65^ respectively, indicating that HCO_3_^–^ and HPO_4_^2–^ are the strongest nucleophiles among the probed anions. As shown in Figure 5c, the anodic peak potential and current of ceMoS_2_ (highlighted zone) in HCO_3_^–^ and HPO_4_^2–^ illustrated an increase in the oxidation propensity of ceMoS_2_. The compiled results suggest that the enhanced oxidative dissolution of ceMoS_2_ in HCO_3_^–^ and HPO_4_^2–^ was likely similar to that of the AgNPs, as the presence of nucleophiles shifted the chemical potential and facilitated oxidation. Therefore, oxidation of ceMoS_2_ by O_2_ was enhanced by strong nucleophiles.

Additionally, the Mo atom was an electron donor, and the S atom was as an electron acceptor in the electronic structure of MoS_2_,^66^ which indicated covalent Mo–S bonding and an electron-deficient Mo center. Moreover, the chemical exfoliation and lithiation process induced sulfur vacancies on the MoS_2_ nanosheets,^67^ thereby rendering the surrounding Mo atoms more electrophilic.^68,69^ The Mo^VI^–O peak in the Mo 3d core-level spectrum of ceMoS_2_ (Figure 1c) indicated the presence of sulfur vacancies.^70^ When potent nucleophiles (HCO_3_^–^ and HPO_4_^2–^/H_2_PO_4_^–^) were present, the formation of Mo^V^ in the ceMoS_2_ (Figure 4e,f) was the result of reduction of MoS_2_ by the nucleophilic anions. The Mo^V^ altered the chemical potential of ceMoS_2_, which was more susceptible to oxidation and subsequent dissolution.

**Scheme 2:**
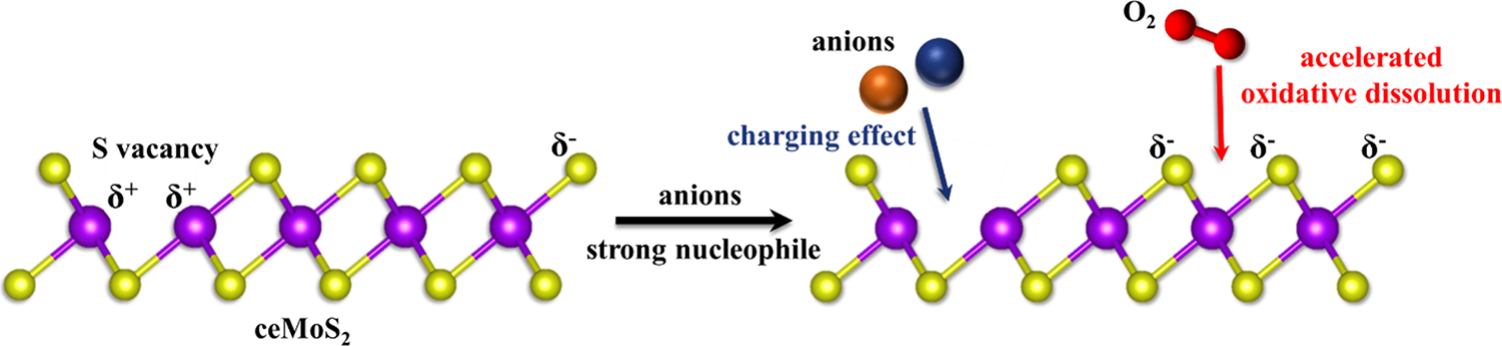
Proposed Mechanism for Oxidative Dissolution of ceMoS_2_ Accelerated by Various Anions

Given that oxidation dissolution of ceMoS_2_ with the electron acceptor O_2_ was catalyzed by nucleophilic anions and involved the electrophilic Mo center, oxygen reduction during the oxidation of ceMoS_2_ was examined by studying the ORR in the presence of different anionic electrolytes. In the ORR polarization curves (Figure 5d), ceMoS_2_ exhibited a more positive onset potential (*E*_onset_) (listed in Table S5) and lower Tafel slope (Figure 5e) in HCO_3_^–^ and HPO_4_^2–^/H_2_PO_4_^–^ compared with other anions. The results indicated that the presence of strong nucleophiles enhanced electron transfer from ceMoS_2_ to oxygen (i.e., faster ORR kinetic process), which was consistent with the higher oxidation current seen for ceMoS_2_ in the presence of HCO_3_^–^ and HPO_4_^2–^/H_2_PO_4_^–^. The proposed mechanism for the effects of anions on ceMoS_2_ is shown in Scheme 2. The presence of anions facilitated the oxidation of ceMoS_2_ by oxygen by triggering changes in the chemical potential of the ceMoS_2_ surface as a function of the nucleophilicities (i.e., charging effect) of the various anions.

### 3.5 Dissolution of ceMoS_2_ under Environmentally Relevant Conditions

To confirm the effects of anions on the MoS_2_ nanosheets, the dissolution of ceMoS_2_ in the presence of anions was surveyed under more environmentally relevant conditions. As listed in Table 2, the initial concentration of dissolved Mo species from the ceMoS_2_ was 12.21 μg/L. After 72 h of incubation in the dark, the *C*_*t*_/*C*_0_ of ceMoS_2_ increased to 2.62, 2.76, and 2.57 for Cl^–^, SO_4_^2–^, and NO_3_^–^, respectively, indicating mild enhancement of ceMoS_2_ dissolution by these three ions. On the other hand, the *C*_*t*_/*C*_0_ for ceMoS_2_ increased substantially to 3.97 and 4.02 in the presence of HCO_3_^–^ and HPO_4_^2–^/H_2_PO_4_^–^, respectively, confirming enhanced promotion of ceMoS_2_ oxidative dissolution. For sunlight irradiation with NO_3_^–^, the *C*_*t*_/*C*_0_ of ceMoS_2_ was 4.94, higher than those seen with Cl^–^ and SO_4_^2–^. These results demonstrated that the impacts of the anions on the stability of the MoS_2_ nanosheets were valid at lower concentrations.

**Table 2:** Concentrations of the Ionic Mo Species (Obtained by Passing ceMoS_2_ Suspensions through 3 kDa Membranes) Produced by ceMoS_2_ in the Presence of Anions under Environmentally Relevant Conditions for 72 h (Initial Concentration: 100 μg/L ceMoS_2_ and 1 mM Anions)

ceMoS_2_ anionic samples	ionic Mo species at 0 h	ionic Mo species at 72 h in dark		ionic Mo species at 72 h under sunlight irradiation	
	*C*_0_ (μg/L)	*C_t_* (μg/L)	*C_t_*/*C*_0_	*C_t_* (μg/L)	*C_t_*/*C*_0_
blank	12.21 ± 0.31	26.76 ± 2.07	2.19 ± 0.22	43.09 ± 1.38	3.53 ± 0.20
Cl^–^		32.05 ± 1.96	2.62 ± 0.23	43.29 ± 2.16	3.55 ± 0.33
SO_4_^2–^		33.71 ± 0.78	2.76 ± 0.13	51.82 ± 1.76	4.24 ± 0.25
NO_3_^–^		31.42 ± 0.28	2.57 ± 0.09	60.28 ± 1.39	4.94 ± 0.24
HCO_3_^–^		48.44 ± 1.02	3.97 ± 0.18	82.88 ± 3.98	6.79 ± 0.50
HPO_4_^2–^/H_2_PO_4_^–^		49.08 ± 1.37	4.02 ± 0.21	69.32 ± 0.83	5.68 ± 0.21

## 4 Environmental Implications

The chemical stability of the MoS_2_ nanosheets has a considerable impact on the potential uses of MoS_2_, especially in aqueous environments. Our findings demonstrated that the oxidative dissolution of ceMoS_2_ was affected by the nucleophilicities of coexisting anionic species. The ceMoS_2_ charging effects of the nucleophilic anions (i.e., HCO_3_^–^ and HPO_4_^2–^/H_2_PO_4_^–^) shifted the chemical potential of ceMoS_2_, promoting oxidative dissolution of the ceMoS_2_. With the fast charging by HCO_3_^–^ and HPO_4_^2–^/H_2_PO_4_^–^, the oxidative transformation of the MoS_2_ nanosheets accelerated the morphology and phase transitions, leading to deteriorated sheets, a 1T to 2H transition, and the emergence of pentavalent Mo (Mo^V^). It is noteworthy that over the past two decades, the average atmospheric CO_2_ concentration has increased from 372.59 ppm in 2002 to 418.64 ppm in 2023, with an annual growth rate of approximately 2 ppm.^71^ Most of the dissolved inorganic carbon (DIC) will likely generate HCO_3_^–^ (>90%) and elevate the concentrations of HCO_3_^–^ in aqueous environments.^72^ Between 2004 and 2019, the DIC level in the global ocean increased from 16 to 38% at depths shallower than 1500 m.^73^ Thus, the reactions of HCO_3_^–^ with released ENMs, including MoS_2_, in aquatic environments hold increasing importance. The effects of anions could also provide insights into the environmental implications of MoS_2_ nanosheets, including their toxicity and bioavailability. It has been shown that the transformed ENMs throughout environmental processes exhibit different toxicity profiles from those of as-prepared materials.^74^ Our prior study demonstrated that acidic ionic Mo species released during the aging process of MoS_2_ nanosheets were detrimental to aquatic organisms.^75^ Given the dependence of ceMoS_2_ oxidative dissolution on the anionic species studied in the present work, the ecological risks of the transformed MoS_2_ nanosheets could be shifted by these species. Additionally, our findings demonstrated the accelerated dissolution of ceMoS_2_ with anions, particularly NO_3_^–^, HCO_3_^–^, and HPO_4_^2–^/H_2_PO_4_^–^, under sunlight exposure. Therefore, with the numerous photocatalytic applications of MoS_2_ in aquatic environments,^76,77^ it is important to consider the presence of anionic species when assessing the durability of MoS_2_ for photocatalytic water treatment.

## Supporting Information Available

Supplementary experimental section; residual molar ratio of Li to Mo and pH values of ceMoS_2_ suspension; correlation between ceMoS_2_ concentrations and absorbance at 450 nm; spectra of natural sunlight and light emitted in the CPS+ solar simulator and transmittance spectrum of the UV-cutoff filter; passing ratio of Na_2_MoO_4_ through the 3 kDa MWCO membranes; IS calculation of ionic species used in this study; effect of Cl^–^ on the normalized absorbance at 450 nm of ceMoS_2_; stabilities of ceMoS_2_ suspensions in the presence of 1 mM anionic species with and without elimination of O_2_; EPR spectra for DMPO adducts in ceMoS_2_ with HCO_3_^–^ and HPO_4_^2–^/H_2_PO_4_^–^; zeta potential measurement of ceMoS_2_ with sodium anionic species; aggregation profiles of ceMoS_2_ suspensions with sodium anionic species; rate constants of absorbance in ceMoS_2_ with coexisting anionic species; EPR spectra for DMPO adducts in NO_3_^–^ under irradiation; UV–vis absorption spectra of sodium anionic species and the spectrum of light emitted by the CPS+ solar simulator; ion chromatograms of ceMoS_2_ incubated with HCO_3_^–^; standard reduction potential of anionic species; ionic radius of anionic species used in this study; pH variation of ceMoS_2_ incubated with anionic species; pH effects on the stability of ceMoS_2_; Mo 3d XPS spectra of ceMoS_2_ in anions; and the ORR onset potential of ceMoS_2_ in anions (PDF)
