# Supplementary material for: Influence of Inorganic Anions on the Chemical Stability of Molybdenum Disulfide Nanosheets in the Aqueous Environment
Source: Environ Sci Technol. 2024 Jan 29;58(5):2490–501. doi: 10.1021/acs.est.3c08278 (PMC10851429; doi:10.1021/acs.est.3c08278)
Supplement: Supplementary file 1 — es3c08278_si_001.pdf [file es3c08278_si_001.pdf]

Supporting Information for

Influence of inorganic anions on the chemical stability of molybdenum disulfide nanosheets in the aqueous environment

Ting-Wei Lee and Chiaying Chen*

Department of Environmental Engineering, National Chung Hsing University, Taichung City 402, Taiwan

*E-mail: chiayingchen@nchu.edu.tw

Number of pages: 24

Number of texts: 1

Number of figures: 15

Number of tables: 5

Contents

Text S1	Supplementary experimental section	Page S3
Figure S1	Residual molar ratio of Li to Mo and pH values of ceMoS$_2$ suspension	Page S4
Figure S2	Correlation between ceMoS$_2$ concentrations and absorbance at 450 nm	Page S5
Figure S3	Spectra of natural sunlight and light emitted in the CPS+ solar simulator and transmittance spectrum of the UV-cutoff filter	Page S6
Figure S4	The passing ratio of Na_2MoO_4 through the 3 kDa MWCO membranes	Page S7
Table S1	IS calculation of ionic species used in this study	Page S8
Figure S5	Effect of Cl^- on the normalized absorbance at 450 nm of ceMoS$_2$	Page S9
Figure S6	Stabilities of ceMoS$_2$ suspensions in the presence of 1 mM anionic species with and without elimination of O_2	Page S10
Figure S7	EPR spectra for DMPO adducts in ceMoS$_2$ with HCO_3^- and $HPO_4^{2-}/H_2PO_4^-$ under irradiation	Page S11
Figure S8	Zeta potential measurement of ceMoS$_2$ with sodium anionic species	Page S12
Figure S9	Aggregation profiles of ceMoS$_2$ suspensions with sodium anionic species	Page S13
Table S2	Rate constants of absorbance in ceMoS$_2$ with coexisting anionic species	Page S14

Figure S10	EPR spectra for DMPO adducts in NO_3^- under irradiation	Page S15
Figure S11	UV-vis absorption spectra of sodium anionic species and the spectrum of light emitted by the CPS+ solar simulator	Page S16
Figure S12	Ion chromatograms of ceMoS_2 incubated with HCO_3^-	Page S17
Table S3	Standard reduction potential of anionic species	Page S18
Table S4	Ionic radius of anionic species used in this study	Page S19
Figure S13	pH variation of ceMoS_2 incubated with anionic species	Page S20
Figure S14	pH effects on the stability of ceMoS_2	Page S21
Figure S15	Mo 3d XPS spectra of ceMoS_2 in anions	Page S22
Table S5	The ORR onset potential of ceMoS_2 in anions	Page S23
References		Page S24

Text S1 Supplementary experimental section

Chemicals

Molybdenum (IV) disulfide (98.5%), *n*-butyllithium (1.6 M in hexane), and sodium nitrate (99%+) were purchased from Acros Organics. Sodium chloride (≥ 99.5%) was obtained from Honeywell. Sodium sulfate (≥ 99%) was from J.T. Baker. Sodium bicarbonate (≥ 99.7%) was from Sigma-Aldrich. Sodium dihydrogen phosphate anhydrous (98%) was purchased from SHOWA. Di-sodium hydrogen orthophosphate dihydrate (≥ 99%) was obtained from Fisher Chemical. 5,5-Dimethyl-1-pyrroline-N-oxide (DMPO, 97%) was from Tokyo Chemical Industry. All sample solutions were prepared by deionized (DI) water (≥ 18.2 Ω·cm).

Preparation of chemical exfoliation MoS_2 nanosheets

The preparation of chemical exfoliation MoS_2 nanosheet solutions (ceMoS_2) followed our previous study with minor adjustments.[1] In short, 300 mg of MoS_2 powder was mixed with 3 mL of 1.6 M *n*-butyllithium and stirred mildly under a nitrogen atmosphere in a glovebox for 2 days. After lithium-intercalation, the black mud was filtered with a 0.22 μm PTFE membrane filter paper (Whatman^{TM}) and rinsed with 20 mL hexane three times. Then, the resulting product was dispersed in DI water straightway by bath ultrasonication for 1 h. The byproduct LiOH was removed by dialysis against DI water for 3 days and followed by centrifugation at 4000 rpm for 15 min. After the dialysis process, the pH value of the suspension declined from approximately 12 to 7, and the Li: Mo ratio decreased from 2.08 to 0.77 (Figure S1), both of which indicated a successful removal of LiOH from the ceMoS_2 suspension.[2, 3] Finally, a dark dispersion of ceMoS_2 was obtained.

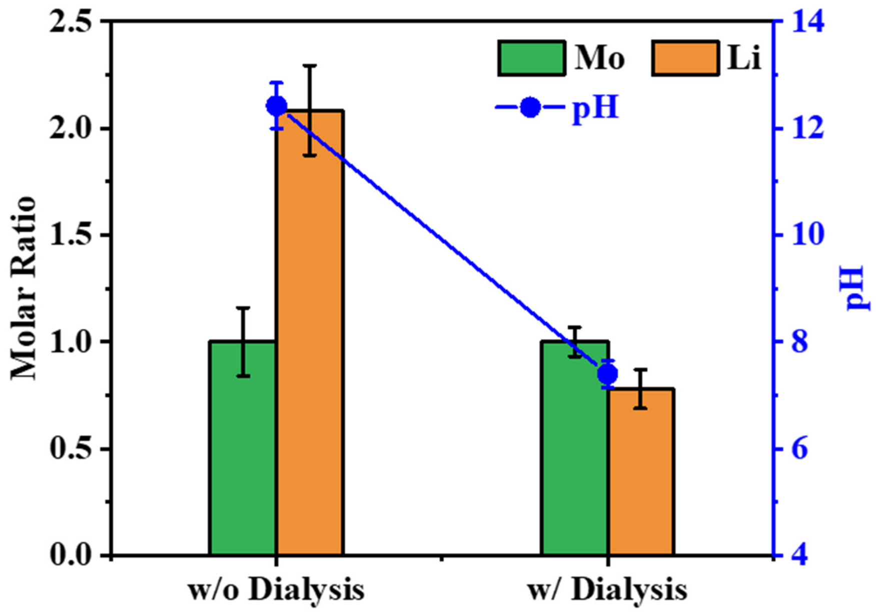

Figure S1. Residual molar ratio of Li to Mo and pH values of $ceMoS_2$ suspension.

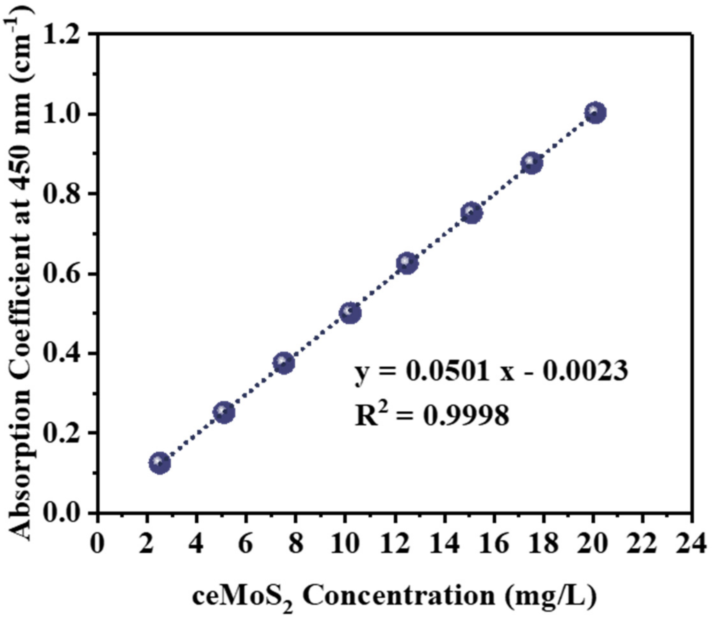

Figure S2. Correlation between $ceMoS_2$ suspension concentrations and the absorbance at 450 nm, showing a linear regression with a mass extinction coefficient of 5010 L m^{-1} g^{-1}.

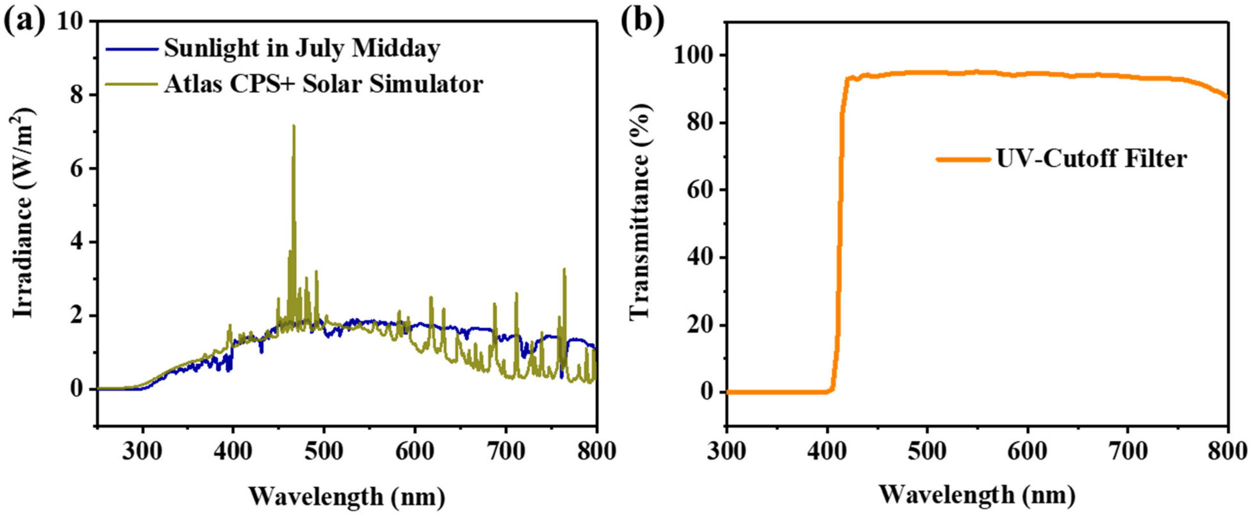

Figure S3. (a) Spectra of natural sunlight and light emitted by the Xenon lamp in the CPS+ solar simulator used in this study. (b) Transmittance spectrum of the UV-cutoff filter, showing the blockage of irradiation with wavelengths below 420 nm.

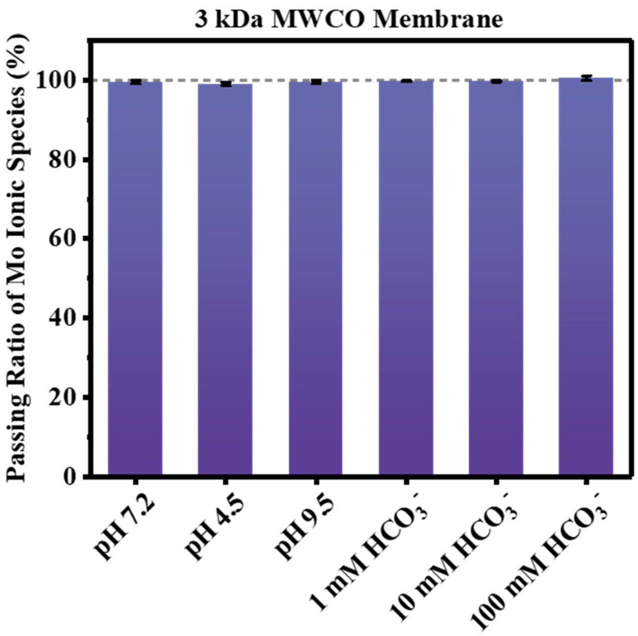

Figure S4. The passing ratio of sodium molybdate solution (initial concentration: 5 mg/L as Mo) through the 3 kDa MWCO membranes utilized, indicating that there is no adsorption loss of Mo ionic species to the membrane (n = 3).

Table S1. Ionic strength (IS) calculation of ionic species used in this study.

Ionic Species	Conc. (mM)	Cation		Anion		IS[a] (mM)
		Charge number	Conc. (mM)	Charge number	Conc. (mM)	
NaCl	1	+1	1	-1	1	1
	2		2		2	2
	5		5		5	5
	10		10		10	10
	20		20		20	20
	100		100		100	100
$NaNO_3$	1	+1	1	-1	1	1
	10		10		10	10
	100		100		100	100
Na_2SO_4	1	+1	2	-2	1	3
	10		20		10	30
	100		200		100	300
$NaHCO_3$	1	+1	1	-1	1	1
	10		10		10	10
	100		100		100	100
NaH_2PO_4/Na_2HPO_4 (ratio: 25.8/74.2)	1.742	+1	1.742	-1/-2	0.258/0.742	2.484
	17.42		17.42		2.58/7.42	24.84
	174.2		174.2		25.8/74.2	248.4

IS[a]: Ionic strength, IS was calculated by $\text{IS (mM)} = \frac{1}{2} \sum m_i \cdot z_i^2$, where m_i is the ionic concentration (mM) and z_i is the number of charges on ions.[4]

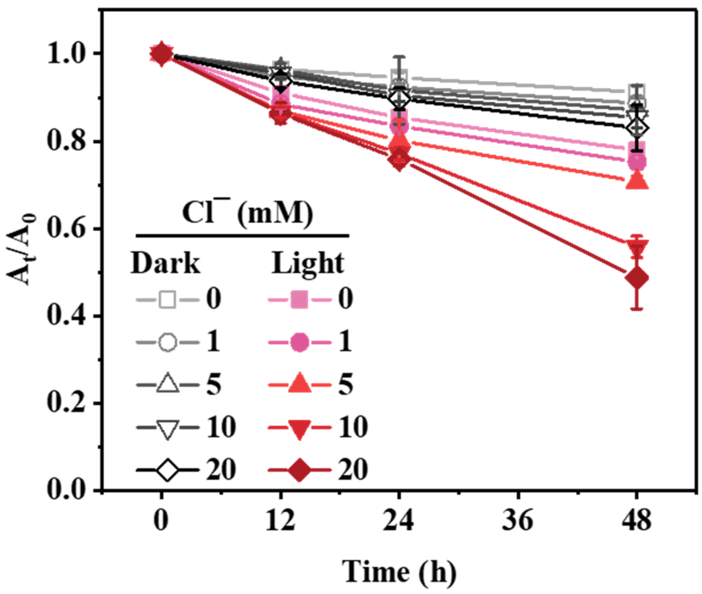

IS (mM)	Ionic Mo species at 0 h	Ionic Mo species at 72 h in dark		Ionic Mo species at 72 h under irradiation	
	mg/L	mg/L	Ionic Mo species production rate (%)	mg/L	Ionic Mo species production rate (%)
0	2.47 ± 0.01	3.19 ± 0.01	29.1 ± 0.9	3.64 ± 0.12	47.4 ± 3.4
1		3.34 ± 0.17	35.2 ± 2.4	3.79 ± 0.12	53.4 ± 3.5
5		3.46 ± 0.11	40.1 ± 2.0	3.92 ± 0.15	58.7 ± 2.8
10		3.53 ± 0.15	42.9 ± 1.6	4.01 ± 0.14	62.3 ± 2.3
20		3.81 ± 0.13	54.3 ± 2.2	4.15 ± 0.17	68.0 ± 2.5

Figure S5. Effect of Cl^- on the normalized absorbance at 450 nm (A_t/A_0) and concentrations of ionic Mo species (samples were obtained by passing through 3 kDa membranes) in ceMoS_2 (10.5 mg/L).

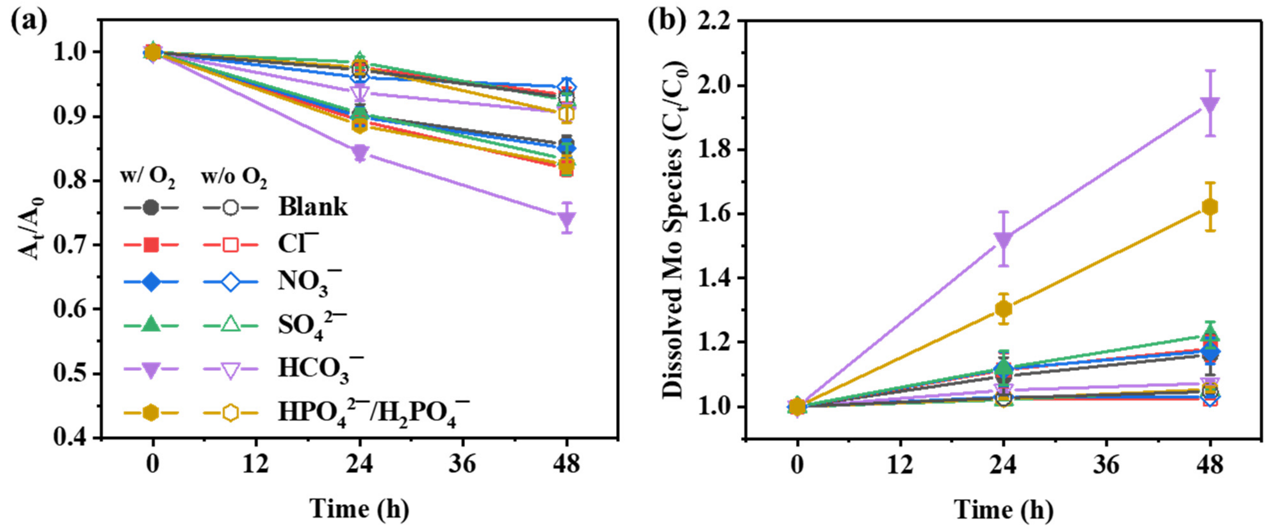

Figure S6. Stabilities of ceMoS$_2$ suspensions (10.5 mg/L) determined by measuring (a) the normalized absorbance at 450 nm (A_t/A_0) and (b) the normalized concentration of dissolved Mo species (C_t/C_0) in the presence of 1 mM anionic species with and without elimination of O_2.

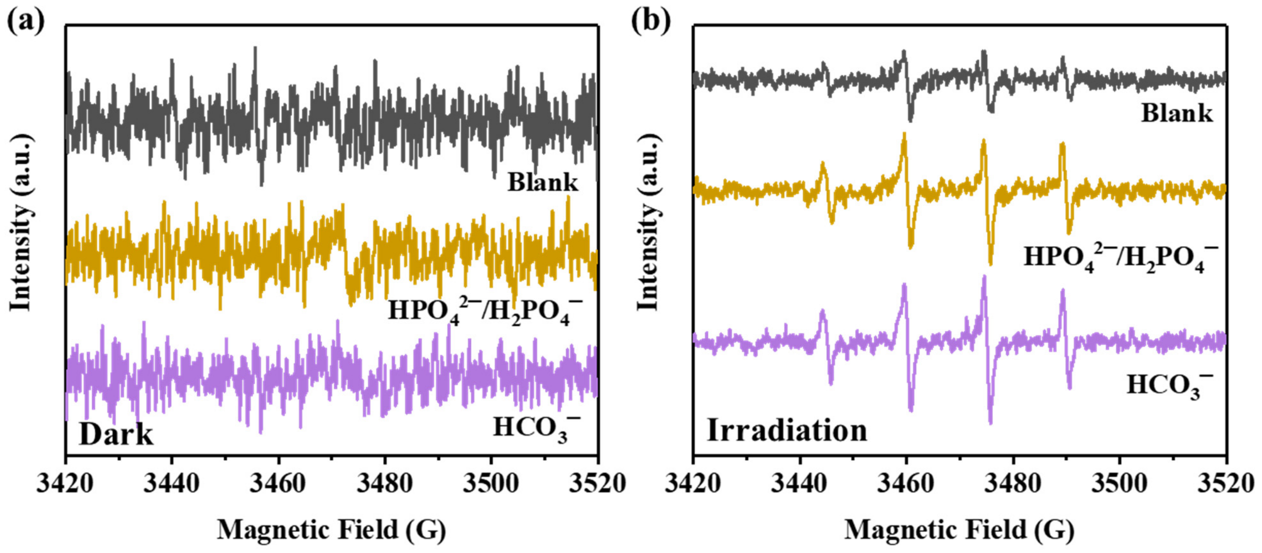

Figure S7. EPR spectra of ceMoS$_2$ suspensions (10.5 mg/L) with or without HCO_3^- and $HPO_4^{2-}/H_2PO_4^-$ (10 mM) under both (a) dark and (b) irradiation, indicating the enhanced •OH generation under irradiation.

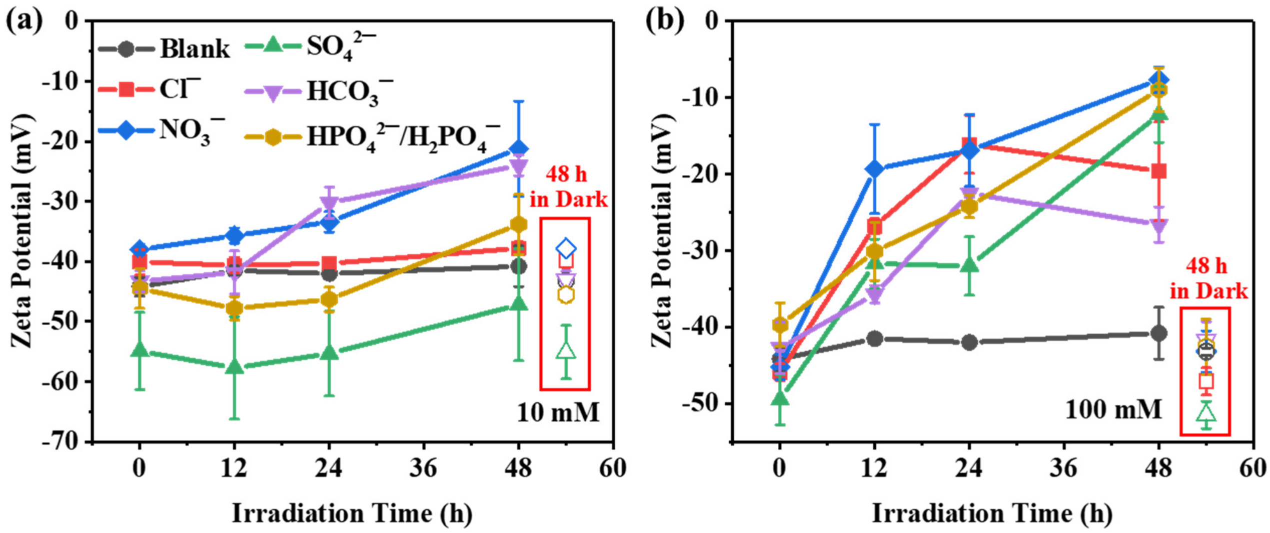

Figure S8. Zeta potential measurement of $ceMoS_2$ suspensions (10.5 mg/L) in the presence of 10 mM and 100 mM anionic species under both dark and solar irradiation.

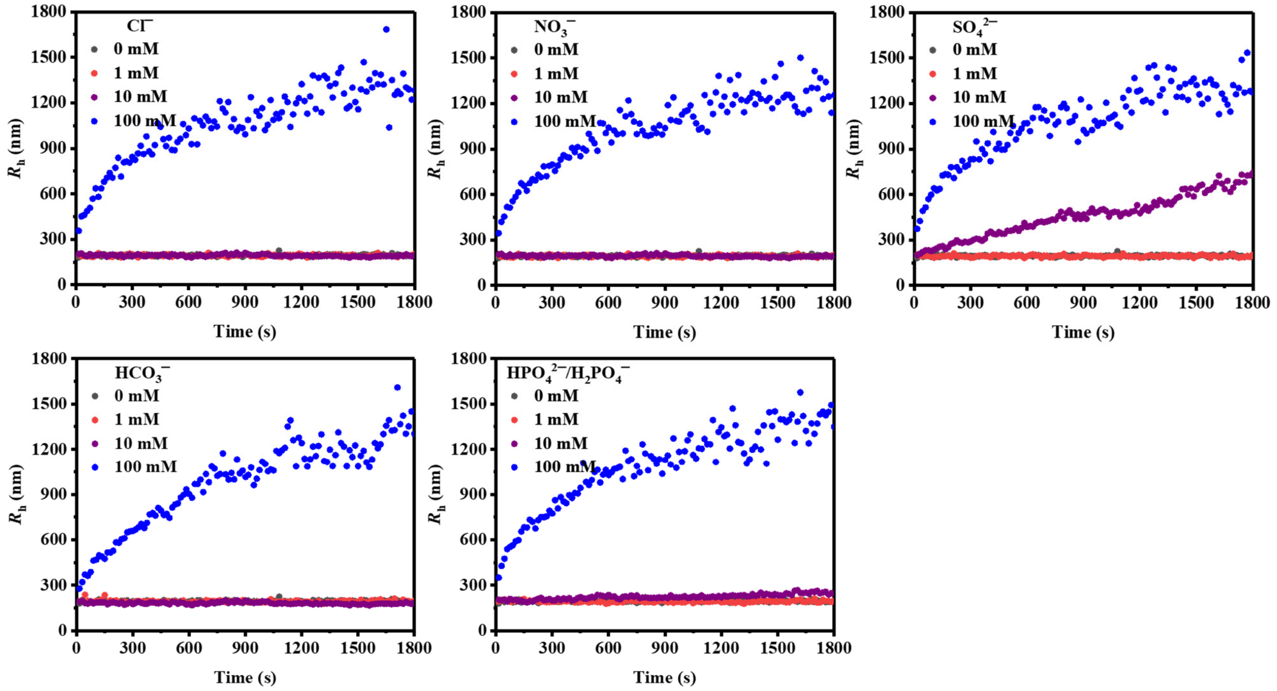

Figure S9. Aggregation profiles of ceMoS2 suspensions (10.5 mg/L) in the presence of 1 mM, 10 mM, and 100 mM anionic species.

Table S2. Rate constants of absorbance at 450 nm in ceMoS$_2$ with coexisting anionic species, computed from data in Figure 2.

Sample	Absorbance (A_t/A_0)			
	Dark		Irradiation	
	k (h^{-1})	R^2	k (h^{-1})	R^2
Blank	0.00351 ± 0.00031	0.9760	0.00551 ± 0.00052	0.9738
1 mM				
Cl^-	0.00443 ± 0.00044	0.9703	0.00630 ± 0.00083	0.9503
NO_3^-	0.00369 ± 0.00037	0.9700	0.00606 ± 0.00077	0.9526
SO_4^{2-}	0.00394 ± 0.00020	0.9916	0.00602 ± 0.00093	0.9331
HCO_3^-	0.00653 ± 0.00043	0.9866	0.02010 ± 0.00026	0.9994
$HPO_4^{2-}/H_2PO_4^-$	0.00435 ± 0.00042	0.9719	0.01032 ± 0.00054	0.9916
10 mM				
Cl^-	0.00513 ± 0.00102	0.8935	0.00944 ± 0.00073	0.9822
NO_3^-	0.00379 ± 0.00045	0.9589	0.01197 ± 0.00044	0.9957
SO_4^{2-}	0.00691 ± 0.00099	0.9411	0.01139 ± 0.00111	0.9723
HCO_3^-	0.00692 ± 0.00052	0.9832	0.02389 ± 0.00065	0.9979
$HPO_4^{2-}/H_2PO_4^-$	0.00514 ± 0.00039	0.9826	0.01474 ± 0.00084	0.9901
100 mM				
Cl^-	0.02336 ± 0.00125	0.9915	0.03301 ± 0.00378	0.9622
NO_3^-	0.02008 ± 0.00066	0.9967	0.04209 ± 0.00788	0.9047
SO_4^{2-}	0.02964 ± 0.00279	0.9740	0.03443 ± 0.00467	0.9477
HCO_3^-	0.03424 ± 0.00309	0.9762	0.04727 ± 0.00602	0.9536
$HPO_4^{2-}/H_2PO_4^-$	0.02480 ± 0.00206	0.9797	0.02989 ± 0.00365	0.9571

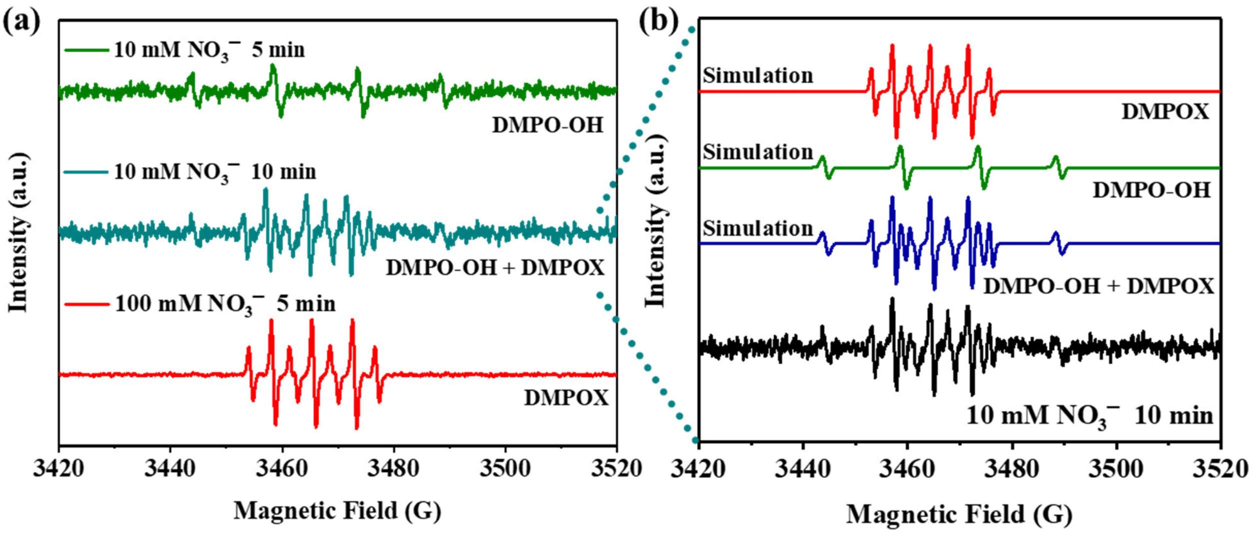

Fitting Parameters for 10 mM NO_3^- 10 min	DMPO-OH	DMPOX
g factor	2.00662	2.00773
Area	0.09938	0.23160
A_H	14.8515	7.25727
A_N	15.0026	4.00997

Figure S10. EPR spectra for DMPO adducts in NO_3^- under irradiation. (a) DMPO-OH and DMPOX signals in 10 and 100 mM of NO_3^- and (b) the simulated DMPO-OH + DMPOX signal, showing the EPR signal of 10 mM NO_3^- after irradiation for 10 min was a mixture of DMPO-OH and DMPOX.

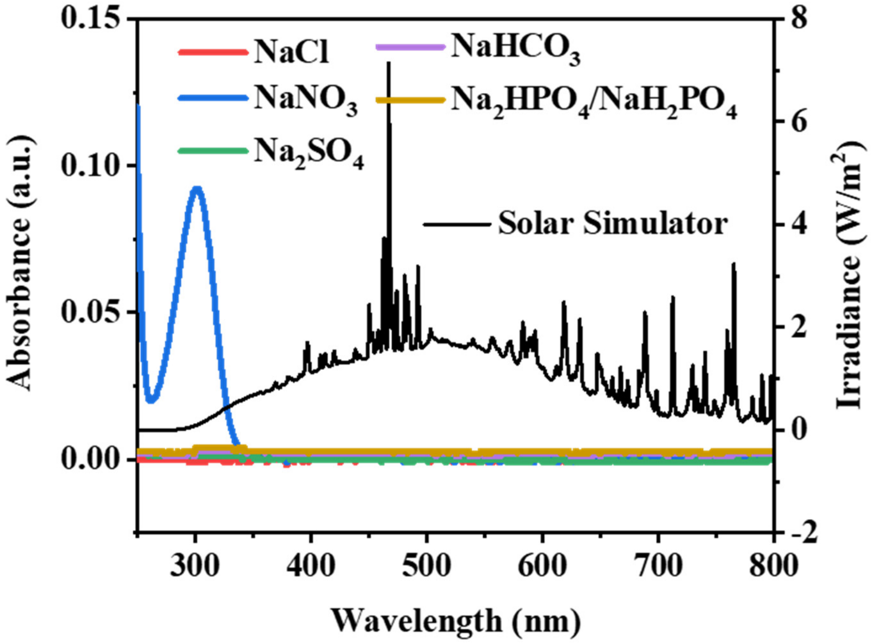

Figure S11. UV-vis absorption spectra of 10 mM sodium anionic species and the spectrum of light emitted by the Xenon lamp in the CPS+ solar simulator, showing the absorption characteristic band of NO_3^- below 350 nm.

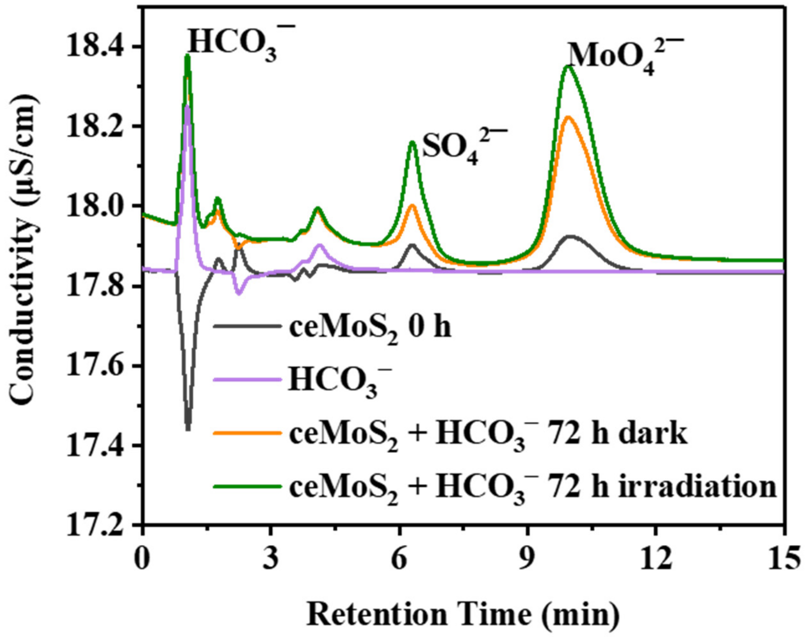

Figure S12. Ion chromatograms of $ceMoS_2$ (10.5 mg/L) incubated with HCO_3^- (10 mM) for 72 h, showing the dissolution of SO_4^{2-} and MoO_4^{2-} from $ceMoS_2$ by HCO_3^-.

Table S3. Standard reduction potential of the reduction half-reaction of anionic species.[5-8]

(Note that chloride is absent in this table since it is the highest reduced state among chlorine species)

Anionic species	Reduction half-reaction	Standard reduction potential (E^0)
Nitrate	$NO_3^- + H_2O + 2e^- \rightarrow NO_2^- + 2OH^-$	0.01 V
	$NO_3^- + 3H_2O + 5e^- \rightarrow 0.5N_2 + 6OH^-$	0.26 V
	$NO_3^- + 6H_2O + 8e^- \rightarrow NH_3 + 9OH^-$	-0.12 V
Sulfate	$SO_4^{2-} + 4H^+ + 2e^- \rightarrow H_2SO_3 + H_2O$	0.172 V
	$2SO_4^{2-} + 4H^+ + 2e^- \rightarrow S_2O_6^{2-} + H_2O$	-0.22 V
	$SO_4^{2-} + H_2O + 2e^- \rightarrow SO_3^{2-} + 2OH^-$	-0.93 V
Bicarbonate	$4HCO_3^- + 28H^+ + 24e^- \rightarrow 4CH_3OH + 8H_2O$	-0.373 V
	$2HCO_3^- + 9H^+ + 8e^- \rightarrow CH_3COO^- + 4H_2O$	-0.279 V
	$HCO_3^- + 9H^+ + 8e^- \rightarrow 4CH_4 + 3H_2O$	-0.259 V
Phosphate	$H_3PO_4 + 2H^+ + 2e^- \rightarrow H_3PO_3 + H_2O$	-0.276 V
	$PO_4^{3-} + 2H_2O + 2e^- \rightarrow HPO_3^{2-} + 3OH^-$	-1.05 V

Table S4. Ionic radius of anionic species used in this study.[9, 10]

Anions	Ionic Radius (pm)
Cl^-	181
NO_3^-	179
SO_4^{2-}	258
HCO_3^-	156
$HPO_4^{2-}/H_2PO_4^-$	200 ($H_2PO_4^-$)<br>145[a] (HPO_4^{2-})

[a]: The ionic radius of HPO_4^{2-} was calculated by $V=\left(\frac{4\pi N_A}{3}\right)r^3$, where V is the conventional partial molar volume of HPO_4^{2-} in water at 25°C (7.7 $cm^3\ mol^{-1}$),[11] N_A is Avogadro constant (6.022×10^{23} mol^{-1}), and r is the ionic radius of HPO_4^{2-}.

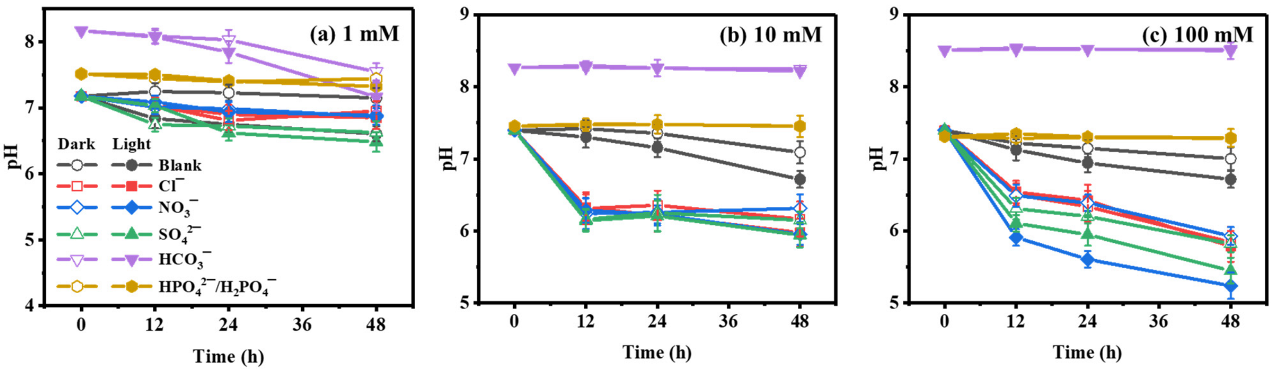

Figure S13. pH variation of ceMoS$_2$ incubated with (a) 1mM, (b) 10 mM and (c) 100 mM of anionic species under both dark and solar irradiation.

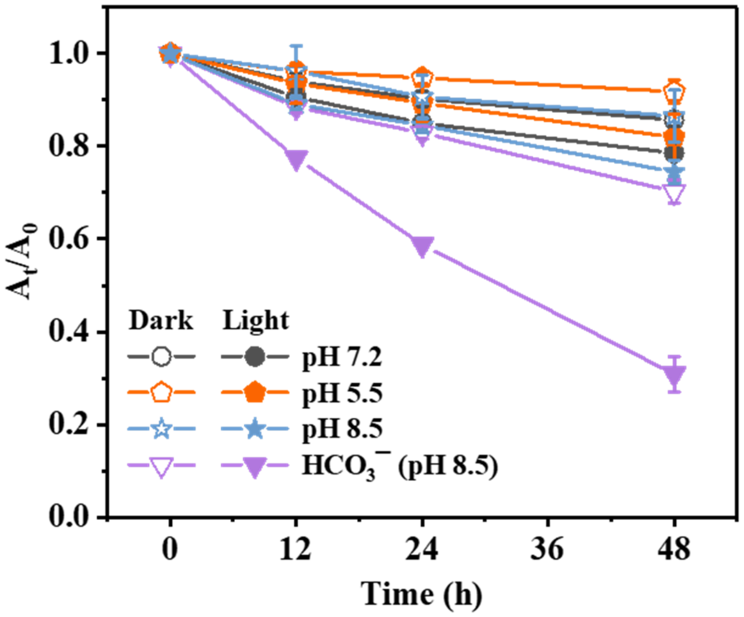

Figure S14. pH effects on the normalized absorbance at 450 nm (A_t/A_0) of $ceMoS_2$ (10.5 mg/L).

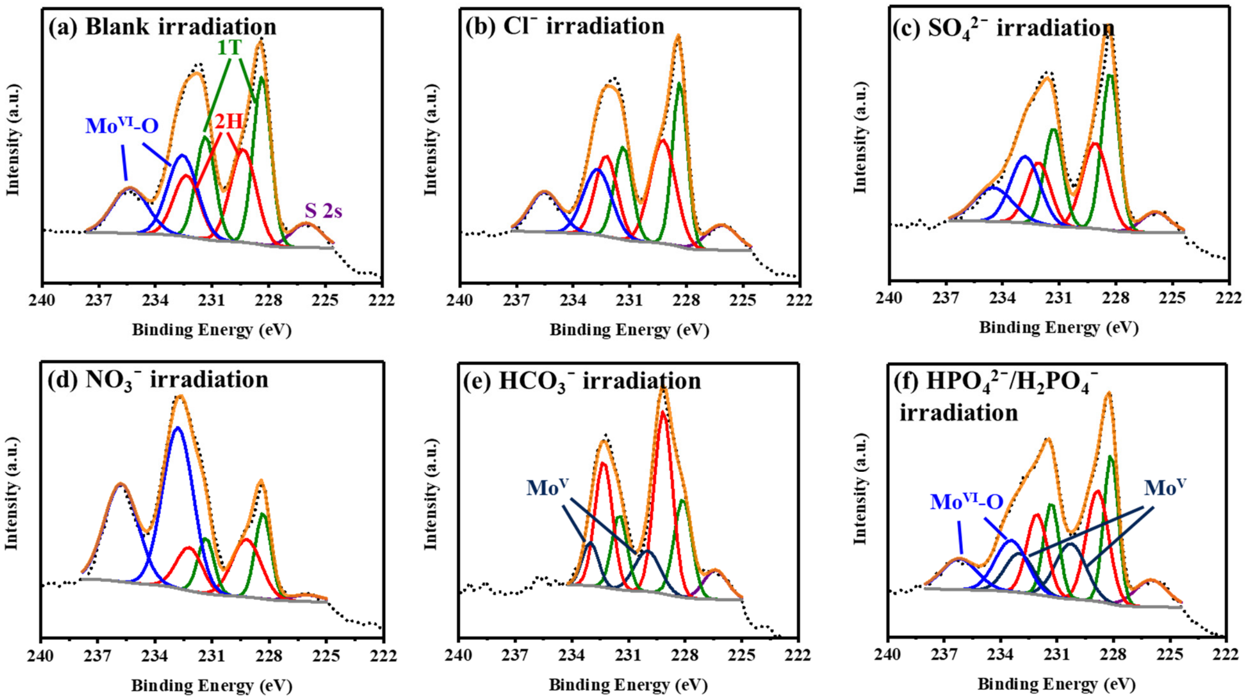

(%)	Blank		Cl^-		SO_4^{2-}		
	Mo^{VI}-O	Mo-S (1T:2H)	Mo^{VI}-O	Mo-S (1T:2H)	Mo^{VI}-O	Mo-S (1T:2H)	
72 h in dark	19.8	80.2 (58.7:41.3)	32.1	67.9 (54.8:45.2)	18.9	81.1 (55.6:44.4)	
72 h under irradiation	30.9	69.1 (55.4:44.6)	26.5	73.5 (48.2:51.8)	27.1	72.9 (52.0:48.0)	
(%)	NO_3^-		HCO_3^-		$HPO_4^{2-}/H_2PO_4^-$		
	Mo^{VI}-O	Mo-S (1T:2H)	Mo^V	Mo-S (1T:2H)	Mo^{VI}-O	Mo^V	Mo-S (1T:2H)
72 h in dark	32.1	67.9 (57.1:42.9)	24.0	76.0 (38.0:62.0)	--	32.4	67.6 (51.4:48.6)
72 h under irradiation	62.0	38.0 (44.6:55.4)	17.2	82.8 (34.4:65.6)	20.3	21.2	58.5 (46.8:53.2)

Figure S15. Mo 3d XPS spectra of $ceMoS_2$ in anions under solar irradiation for 72 h and the XPS fitting data of $ceMoS_2$ in anions under both under dark and irradiation.

Table S5. The ORR onset potential (E_{onset}) of ceMoS$_2$ in anions (100 mM).

Anions	E_{onset} (V vs. *RHE*)
Cl^-	0.359
NO_3^-	0.332
SO_4^{2-}	0.350
HCO_3^-	0.413
$HPO_4^{2-}/H_2PO_4^-$	0.425

References

1 Lee, T.-W.; Chen, C.-C.; Chen, C., Chemical stability and transformation of molybdenum disulfide nanosheets in environmental media. *Environ. Sci. Technol.*, **2019**, *53*, (11), 6282-6291.

2 Chou, S. S.; De, M.; Kim, J.; Byun, S.; Dykstra, C.; Yu, J.; Huang, J.; Dravid, V. P., Ligand conjugation of chemically exfoliated MoS_2. *J. Am. Chem. Soc.*, **2013**, *135*, (12), 4584-4587.

3 Heising, J.; Kanatzidis, M. G., Exfoliated and restacked MoS_2 and WS_2: Ionic or neutral species? Encapsulation and ordering of hard electropositive cations. *J. Am. Chem. Soc.*, **1999**, *121*, (50), 11720-11732.

4 Solomon, T., The definition and unit of ionic strength. *Journal of Chemical Education*, **2001**, *78*, (12), 1691.

5 Haynes, W. M.; Lide, D. R.; Bruno, T. J., CRC handbook of chemistry and physics. CRC press: **2016**.

6 Szpyrkowicz, L.; Daniele, S.; Radaelli, M.; Specchia, S., Removal of NO_3^- from water by electrochemical reduction in different reactor configurations. *Applied Catalysis B: Environmental*, **2006**, *66*, (1), 40-50.

7 Jin, Q.; Kirk, M. F., Thermodynamic and kinetic response of microbial reactions to high CO_2. *Frontiers in Microbiology*, **2016**, *7*, 1696.

8 Yang, F.; Zhang, C.; Rong, H.; Cao, Y., Research progress and application prospect of anaerobic biological phosphorus removal. *Applied Microbiology and Biotechnology*, **2019**, *103*, (5), 2133-2139.

9 Li, W.-K.; Zhou, G.-D.; Mak, T., Advanced structural inorganic chemistry. Oxford University Press: **2008**.

10 Marcus, Y., Thermodynamics of solvation of ions. Part 5.—Gibbs free energy of hydration at 298.15 K. *Journal of the Chemical Society, Faraday Transactions*, **1991**, *87*, (18), 2995-2999.

11 Millero, F. J., Molal volumes of electrolytes. *Chemical Reviews*, **1971**, *71*, (2), 147-176.
